# Quantifying the Sustainability of Water Availability for the Water‐Food‐Energy‐Ecosystem Nexus in the Niger River Basin

**DOI:** 10.1029/2018EF000923

**Published:** 2018-09-22

**Authors:** Jie Yang, Y. C. Ethan Yang, Hassaan F. Khan, Hua Xie, Claudia Ringler, Andrew Ogilvie, Ousmane Seidou, Abdouramane Gado Djibo, Frank van Weert, Rebecca Tharme

**Affiliations:** ^1^ State Key Laboratory of Eco‐hydraulics in Northwest Arid Region of China Xi'an University of Technology Xi'an China; ^2^ Department of Civil and Environmental Engineering Lehigh University Bethlehem PA USA; ^3^ Department of Civil and Environmental Engineering University of Massachusetts Amherst Amherst MA USA; ^4^ International Food Policy Research Institute Washington DC USA; ^5^ G‐EAU, AgroParisTech, Cirad, IRD, IRSTEA, Montpellier SupAgro, Université de Montpellier Montpellier France; ^6^ Department of Civil Engineering University of Ottawa Ottawa Ontario Canada; ^7^ United Nations University Institute for Water, Environment and Health Hamilton Ontario Canada; ^8^ Wetlands International Bamako Mali; ^9^ Wetlands International Wageningen Netherlands; ^10^ Riverfutures Derbyshire UK

**Keywords:** agent‐based modeling, Niger River Basin, reliability, vulnerability, resilience, trade‐offs

## Abstract

Water, food, energy, and the ecosystems they depend on interact with each other in highly complex and interlinked ways. These interdependencies can be traced particularly well in the context of a river basin, which is delineated by hydrological boundaries. The interactions are shaped by humans interacting with nature, and as such, a river basin can be characterized as a complex, coupled socioecological system. The Niger River Basin in West Africa is such a system, where water infrastructure development to meet growing water, food, and energy demands may threaten a productive and vulnerable basin ecosystem. These dynamic interactions remain poorly understood. Trade‐off analyses between different sectors and at different spatial scales are needed to support solution‐oriented policy analysis, particularly in transboundary basins. This study assesses the impact of climate and human/anthropogenic changes on the water, energy, food, and ecosystem sectors and characterizes the resulting trade‐offs through a set of generic metrics related to the sustainability of water availability. Results suggest that dam development can mitigate negative impacts from climate change on hydropower generation and also on ecosystem health to some extent.

## Key Points

- Dynamic interactions among water, food, energy, and ecosystem are evaluated using a two‐way coupled agent‐based SWAT model
- Generic metrics are developed to evaluate the sustainability of water availability for food, energy, and riverine ecosystems simultaneously
- Trade‐offs among different countries and regional sample cases are fully analyzed

## 1 Introduction

Supplying sufficient water, food, and energy while maintaining environmental sustainability is a growing challenge due to rapid population growth, changing lifestyles, ecosystem degradation, increasing water scarcity, political rather than analysis‐based, cross‐sectoral decision‐making, and an uncertain future climate (World Bank, 2016; World Economic Forum, 2017). Many West African countries are facing such challenges as the population in the region is expected to increase almost threefold between 1990 and 2030 to reach 516 million people (United Nations, 2015). At the same time, climate change threatens crop production and food security (Jalloh et al., 2013; Sultan & Gaetani, 2016). The limited economic and institutional capacity in the region renders the situation particularly challenging. The transboundary Niger River Basin is vast, flowing through four, but draining runoff from nine West African countries. Local ecosystem health is fragile and adversely affected by growing water and land degradation, while at the same time governments are pushing for large reservoir development to meet growing water supply, food, and energy needs as well as to control the high interannual variability in water availability.

Several previous modeling studies have evaluated water, food, energy, and environmental issues in the Niger River Basin. Several models focused on the Upper Niger Basin (Angelina et al., 2015; Eisner et al., 2017; Liersch et al., 2013; Neal et al., 2012; Passchier et al., 2005; Picouet et al., 2001; Vetter et al., 2017), while others covered the entire basin (Aich et al., 2014; BRLi and DHI, 2007; Dezetter et al., 2008; Li et al., 2005; Pedinotti et al., 2012; Schuol et al., 2008; Sheffield et al., 2014; Sogreah, 1985). These modeling studies provide valuable assessments of hydrological changes resulting from climatic variations and dam development. Notably, BRLi and DHI (2007) appraised variations in annual runoff using the MIKE BASIN model of the Niger Basin Authority (NBA) and assessed the influence of these variations on hydropower and crop production. Aich et al. (2014) and Koch et al. (2013) evaluated the impacts of climate change on streamflow and identified adaptation options for the Upper Niger Basin with the Soil and Water Integrated Model. Liersch et al. (2013) used the same model to assess crop production and ecosystem vulnerability under climate change and human activities (population growth and water infrastructure development) for the Upper Niger Basin. Angelina et al. (2015), Eisner et al. (2017), and Vetter et al. (2017) used a multimodel approach to assess the uncertainties in the choice of climate and rainfall‐runoff models on projected streamflow parameters in the upper Niger Basin.

In parallel, several studies highlighted the important relationships shaped by the Inner Niger Delta, the largest river floodplain in West Africa and a designated Ramsar wetland site with important associated ecosystem services (Ogilvie et al., 2010, 2015). Kuper et al. (2003) developed an integrated model to characterize the Inner Niger Delta's ecosystem under multiple scenarios that take into account population, flooding, and water infrastructure development. Ghile et al. (2014) explored impacts of climate variation on water infrastructure investment via a risk‐based model. Sidibé and Williams (2016) evaluated the use of water pricing for economic benefits and ecosystem protection using a bioeconomic model.

Most of these previous studies focused on a single sector or bilateral relationships such as water‐food, water‐energy, or water‐ecosystems. Moreover, most were limited to parts of the Niger River Basin (generally either the Upper Niger or the Inner Niger Delta). Comprehensive sustainability assessments that fully consider water, crop production, hydropower, and riverine ecosystem conditions for the entire basin have yet to be developed. Without taking all water‐using sectors and the entire basin geography into account, negative impacts such as declines in riverine ecosystem health as a result of water infrastructure, food production, and energy sector developments might be underappreciated or missed (Bhaduri et al., 2015; Bizikova et al., 2013; Karabulut et al., 2016; Lawford et al., 2013; Scott et al., 2011). Also, previous studies failed to assess trade‐offs among different regions in the Niger River Basin. However, trade‐off analysis, including between regions, is essential in the study of transboundary nexus problems (Bhaduri et al., 2015; Scott et al., 2011). To fully assess the sustainability and potential trade‐offs at basin‐wide, national, and regional levels, a comprehensive modeling framework representing both natural processes and human behavior is needed to explicitly quantify the dynamic interactions among water, food, energy, and supporting ecosystems.

A standardized method to quantify the sustainability of different sectors is a crucial basis for cross‐sectoral comparisons. Water‐using sectors are closely interlinked, and thus, feedbacks are highly dynamic (Bhaduri et al., 2015). For instance, energy is necessary to distribute water for crop production, while water is needed for cooling in many energy‐generation processes (Bazilian et al., 2011; Cai et al., 2018; Ringler et al., 2013). Any changes in demands for water‐related resources will alter the natural flow regime and affect the aquatic ecosystem. Therefore, quantifying the sustainability of the nexus or even that of individual sectors is challenging (Bazilian et al., 2011). For example, as stated by the hydropower sustainability assessment protocol (http://www.hydrosustainability.org/), the sustainability of dams should consider environmental, social, economic, and technical aspects. Similarly, sustainable intensification of agricultural systems calls for more efficient use of energy and land and water use in agricultural production. Of the four sectors (water, energy, food, and ecosystems), water is the most susceptible to changes in climate, and those changes propagate to other sectors (Bell et al., 2014; Cai et al., 2018). Also, it is easier to import food and energy into river basins than it is to transfer water (Cai et al., 2018) or restore ecosystem health. Therefore, this study aims to analyze the sustainability of and tradeoffs across the water‐food‐energy‐ecosystem nexus from the perspective of water (Bazilian et al., 2011), with this paper being a first, small step toward addressing nexus sustainability challenges. Numerous studies have proposed sustainability metrics in the food system (Defra, 2009; Feenstra, 2005; International Food Policy Research Institute, 2015), the energy system (Afgan et al., 2000; Santoyo‐Castelazo & Azapagic, 2014), and the water system (Shilling et al., 2014; Vollmer et al., 2016). However, most of the proposed indicators such as the *carbon dioxide environmental indicator* for energy (i.e., the amount of carbon dioxide in tons produced by the plant divided by the energy produced in a lifetime), the *nutrition supply diversity indicator* for food, and the *return flow rate indicator* for water were designed for specific sectors and would not be suitable for a system‐wide water sustainability analysis. Therefore, generic metrics that can simultaneously represent the sustainability of water availability for food, energy, and ecosystems are needed.

In order to address these issues, this study (1) develops a set of generic metrics that can be used for water sustainability analysis for the three water‐using sectors (food, hydropower, and ecosystem) and (2) applies an innovative, coupled modeling framework to evaluate water availability for crop production, hydropower generation, and riverine ecosystem health in the Niger River Basin. The modeling framework consists of an agent‐based model (ABM; Yang et al., 2009, 2012) and a process‐based hydrological model, the Soil and Water Assessment Tool (SWAT; Arnold et al., 1998), combined as the ABM‐SWAT model. Agent‐based modeling was selected for its enhanced representation of heterogeneous stakeholders in large transboundary river basin settings. ABM allows the incorporation of differing levels of cooperation among diverse decision‐makers in the basin. While similar studies have been undertaken in East Africa and South Asia (Khan et al., 2017; Yang et al., 2014, 2016; Yang & Wi, 2018), to the best of our knowledge, this is the first study that incorporates water, food (irrigated crops), energy (hydropower), and environmental concerns into a single modeling framework and applies it to the entire Niger River Basin. It is also the first study that attempts to quantify the sustainability of water availability in a conceptually coherent way for trade‐offs analysis (at basin‐wide, national, and regional levels) to support investment decisions.

The paper is organized as follows: section 2 provides a brief description of key characteristics of the Niger River Basin and the various development pathways assessed. Section 3 introduces the methodology to identify the general water sustainability metrics and introduces the coupled ABM‐SWAT modeling framework. Section 4 presents results for the sustainability metrics chosen (reliability, vulnerability, and resilience) under different modeling scenarios. Section 5 discusses the study limitations and potential future work, and Section 6 concludes the paper.

## 2 Study Area and Scenarios Identified

### 2.1 The Niger River Basin

**Figure 1:**
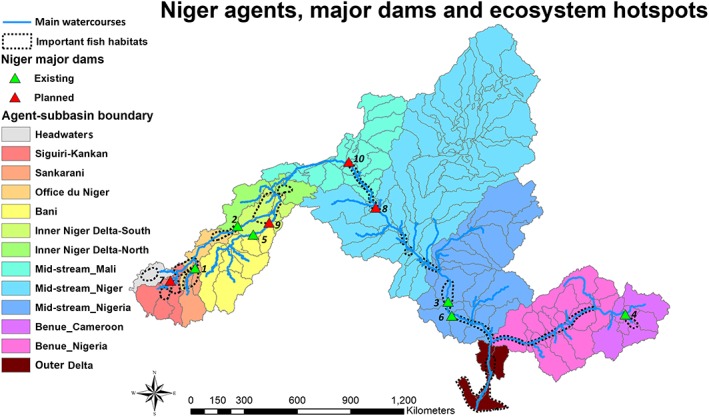
The locations of agents, subbasins, dams, and ecosystem hot spots (important fish habitats) in the Niger River Basin.

The Niger River Basin is the world's ninth largest catchment, with a drainage area of about 2,156,000 km^2^ (Aich et al., 2016). Headwaters of the Niger River system are located in Guinea, and from there the river flows into Mali, through the Inner Niger Delta and then through Niger. In Nigeria, it is joined by its major tributary, the Benue River, before draining into the Atlantic Ocean through the coastal Outer Niger Delta (Figure 1). All nine basin countries fall in the bottom quartile of national economics with respect to gross domestic product (GDP; Ogilvie et al., 2010). Agriculture is the sector employing the majority of the population in all basin countries and contributes up to 40% of basin‐wide GDP. The main crops produced are corn, millet, and sorghum. Increasing energy demand due to a growing population, rapid urbanization, and industrialization is a basin‐wide concern (Bhattacharyya et al., 2015). To satisfy the growing demand for water resources in this fragile ecosystem, the NBA Investment Program and Operational Plan explicitly highlights the development of large‐scale infrastructure (NBA, 2012).

Such infrastructure includes the ongoing and proposed construction of large dams, notably the Fomi, Touassa, and Kandadji dams, along the Niger River and its tributaries (Table 1). It also encompasses the expansions of irrigated agriculture infrastructure across several agricultural development zones (e.g., the Office du Niger in Mali) of the Niger Basin. These water infrastructure projects might be able to mitigate negative climate change effects on crop production and electricity generation to some extent. Meanwhile, such proposed water infrastructure development has raised valid concerns about the conservation of the river system's ecological health, ecosystem services, and biodiversity, as reflected in NBA reports (NBA, 2007), international nongovernmental organization documents (e.g., Wymenga et al., 2011), and the scientific literature (e.g., Rebelo et al., 2013). Water availability and the seasonal flow dynamics of this flood pulse‐driven river system, upon which many wetlands of tremendous socioeconomic and ecological significance depend (Zwarts et al., 2009), might be influenced by hydropower development and extensive irrigation schemes (Goulden et al., 2011). These concerns are highlighted as the second priority field in the NBA Operational Plan, under the rubric of *ecosystem conservation and resources protection*. Thus, the Niger River Basin faces the dual challenges of water infrastructure development to support growing water, food, and energy demand and maintain healthy environmental conditions.

**Table 1:** Characteristics of the 10 Major Niger Basin Dams Modeled

Dam name	Country	Status	Full storage (MCM)	Dead storage (MCM)	Installed hydropower capacity (MW)	Location ID in Figure 1
Selingue	Mali	Existing	2,630	470	44	1
Markala	Mali	Existing	0	0	15	2
Kainji	Nigeria	Existing	15,000	3,000	15,000	3
Lagdo	Cameroon	Existing	6,000	1,450	6,000	4
Talo	Mali	Existing	180	14	N/A	5
Jebba	Nigeria	Existing	3,880	1,000	3,880	6
Fomi	Guinea	Planned	6,160	670	6,160	7
Kandaji	Niger	Planned	1,597	38	1,600	8
Djenne	Mali	Planned	357	60	186	9
Touassa	Mali	Planned	3,150	453	3,150	10

*Note*: Adapted from Koch et al. (2013) and Taner (2017). Abbreviations: MCM = million cubic meters; MW = megawatts; N/A = not applicable.

### 2.2 Scenarios

To evaluate the sustainability of water availability for crop production, hydropower generation, and ecosystem health in the Niger River Basin under different development pathways and external impacts, we tested different scenarios as combinations of different drivers. We followed the concept of *ex ante scenario analysis* (Brown et al., 2012; Lempert & Collins, 2007; Yang et al., 2016), which facilitates the analysis and evaluation of a larger number of climate change and socioeconomic developments (Liersch et al., 2013). Temperature (*T*) and precipitation (*P*) changes were considered as climatic drivers, and alternative water infrastructure developments were considered as socioeconomic drivers in the Niger River Basin. For the climate future assessment, we performed a climate stress test evaluating four levels of temperature change (+0, +1.5, +3, and +4.5 °C) and five levels of precipitation change (+0%, +15%, +30%, −15%, and −30%) based on the historical daily record from 1985 to 2010. Therefore, 20 different climatic conditions (4 × 5) of 26 years of daily temperature and precipitation were evaluated. The downscaled outputs of seven Regional Climate Models for Representative Concentration Pathways 4.5 and 8.5 were then used to inform the likelihood of future climate conditions.

Ten key dams were chosen on the basis of their potential to significantly alter the hydrological regime and change system functional connectivity and health. Six of these dams are operational and four are in the planning or construction stages (Table 1; adapted from Koch et al., 2013, and Taner, 2017). The water infrastructure development scenarios are divided into three different options: (1) the six key existing dams (the baseline scenario), (2) three proposed dams (Djenne, Touassa, and Kandaji dams) that are constructed, and (3) all four proposed dams (Djenne, Touassa, Kandaji, and Fomi dams) that are constructed. We separated the Fomi dam in our analysis because there is an ongoing debate about its implications (e.g., Taner, 2017; Zwarts et al., 2006). The Fomi Dam is the most upstream dam within the basin and has the largest storage and power generation capacity among the proposed dams, and thus, the operation of the Fomi Dam has the potential to markedly change the hydrological regime and ecological character of the Upper Niger River system and the Inner Niger Delta. A total of 60 scenarios (20 climatic × 3 water infrastructure development scenarios) was thus applied in this study.

## 3 Methodology

This section introduces generic metrics that were used to evaluate the sustainability of water availability for three water‐dependent systems (food, energy, and riverine ecosystem health). Additionally, it describes the coupled ABM‐SWAT modeling framework that explicitly models natural processes and human behavior.

### 3.1 Generic Metrics of the Sustainability of Water Availability for Food, Energy, and Riverine Ecosystem

Generic sustainability metrics are indispensable to evaluate the trade‐offs between different regions and across different sectors. For the water‐food‐energy‐ecosystem nexus, decision‐making processes grounded in sustainability should consider the dynamics among different sectors and balance trade‐offs among them. In this case, development of generic metrics that can quantitatively define and measure the sustainability of water availability for food, energy, and riverine ecosystem simultaneously is critical and one of the main purposes of this study. Hashimoto et al. (1982), Loucks (1997), and Sandoval‐Solis et al. (2010) suggested that a quantitative description of the sustainability of water availability should consider three aspects: reliability, resilience, and vulnerability, using a *deficit* concept. In this paper, we apply these three sustainability metrics to agricultural systems (with a focus on irrigated crop production), energy systems (with a focus on hydroelectricity generation), and ecosystems (with a focus on general riverine ecosystem health). A deficit refers here to the difference between target and actual production or between resource demand and supply. We use an annual time step to calculate the sustainability of the three water‐using sectors. Accordingly, we defined annual targets for irrigated crop production, hydropower, and riverine ecosystem health. Therefore, seasonal, monthly, or daily demands of hydropower generation and river ecosystem health were not considered. Crop production and hydropower generation targets are long‐term average annual irrigated crop yields (calculated using the calibrated SWAT model) and annual energy consumption supplied by hydropower (calculated from the daily streamflow simulated by the calibrated SWAT model), respectively. The SWAT model was calibrated and validated with 1985 to 2010 daily streamflow data gathered from the Directions Nationales de l'Hydraulique, the NBA, and the Global Runoff Data Centre.

The target values for each riverine ecosystem hot spot indicated reasonable flow ranges to keep the river healthy. Flow exceeding the upper limit or below the lower limit was considered to lead to the degradation of riverine ecosystem health. For riverine ecosystem functioning, we selected 19 fish biodiversity and fishery hot spots determined by the World Wildlife Fund and the NBA (NBA, 2007). These are concentrated in the Middle Niger‐Benue and in the Upper Niger Basin. All of the 19 hot spots, including the Inner Niger Delta and the Outer Niger Delta, are critical hydroecological regions regarding fish species diversity and associated attributes such as habitat structure and ecological processes. A wide variety of hydroecological indicators can be used as targets for the management of these hot spots. However, because of severe data limitations at the basin scale, we used general, easily computable, ecologically relevant flow indices in the model, namely, Indicators of Hydrologic Alteration (Mathews & Richter, 2007; Richter et al., 1996). We selected the ecologically relevant 7‐day minimum flow as an indicator of low flow conditions and riverine ecosystem health. Given that we used the annual time step to present our results, we selected the smallest value of 7‐day minimum flow during a year as the indicator. The target (i.e., the reasonable range) was set to 0.5 to 5 times the 7‐day minimum flow during a year. The long‐term streamflow was simulated by a calibrated SWAT model. These settings for ecosystem health targets can be readily adapted to include other riverine ecosystem indicators as more quantitative relationships between streamflow regime, wetland hydrodynamics, and ecosystem health are derived across the basin. For instance, for the Inner Niger Delta hot spot, the annual flood maximum discharge is considered a useful indicator of fishery catch, as is the extent of inundation of floodplain habitat (Zwarts et al., 2009). For many other riverine ecosystem services and/or parts of the basin, relationships remain weakly developed and precautionary targets may need to be adopted (e.g., Richter et al., 2012). It is worth noting that irrigated crop production, hydropower generation, and riverine ecosystem functioning targets are kept at current levels despite the expansion in the number of dams and irrigation infrastructure.

In this study, the deficits for the food and energy sectors were calculated with equation (1). We consider both insufficient and excess values as deficit. Retaining minimum flows is essential for the survival of aquatic ecosystems; complete drying up of wetlands and river beds can lead to almost irreversible degradation. However, seasonal low flow conditions are important for triggering and accommodating certain ecological processes, and hence, not having these lower flows could also (but to a lesser extent) lead to ecosystem health decline. Equation (1) was used to calculate the insufficient flow deficit, and Equation (2) was used to calculate the excess flow deficit.

(1) $$D_{t}^{i}=\left\{\begin{array}{c} Q_{\text{target},t}^{i}-Q_{\text{actual},t}^{i}\;\text{if}\;Q_{\text{target},t}^{i}>Q_{\text{actual},t}^{i}\\ 0\;\text{if}\;Q_{\text{target},t}^{i}\leq Q_{\text{actual},t}^{i}\; \end{array}\right.$$

(2) $$D_{t}^{i}=\left\{\begin{array}{c} Q_{\text{actual},t}^{i}-Q_{\text{target},t}^{i}\;\text{if}\;Q_{\text{actual},t}^{i}>Q_{\text{target},t}^{i}\\ 0\;\text{if}Q_{\text{actual},t}^{i}\leq Q_{\text{target},t}^{i}\; \end{array}\right.$$

where $D_{t}^{i}$ is the deficit of different sectors (i.e., food, energy, or riverine ecosystem health) from user *i* at time *t* (while the starting time is *t*_0_ and the total number of time steps is *T*), *Q*_target_ is the targeted amount of water, energy, or food that should be supplied, and *Q*_actual_ is the amount of water, energy, or food that is actually supplied. After the deficit from user *i* has been computed for *T*, three sustainability metrics, reliability (*Rel*), resilience (*Res*), and vulnerability (*Vul*) of individual water‐using sectors, were calculated from this deficit. Reliability represents the probability of the deficit being zero (equation (3); adapted from Hashimoto et al., 1982). Two resilience metrics were calculated. Resilience 1 (*Res*_1_) represents the probability of recovering from a deficit (equation (4); adapted from Loucks, 1997, and Sandoval‐Solis et al., 2010). Resilience 2 (*Res*_2_) represents the deficit recovery rate obtained by integrating functionality, defined as the ratio between actual production and target production ( $Q_{\text{actual},t}^{i}/Q_{\text{target},t}^{i}$) over time (equation (5)). This is also defined as the *resilience triangle* by Bocchini et al. (2013). Vulnerability stands for the degree of severity of a deficit occurrence (equation (6); adapted from Loucks, 1997, and Sandoval‐Solis et al., 2010).

(3) $${\mathrm{Rel}}^{i}=\frac{\mathrm{No}.\text{of times}\;D_{t}^{i}=0\;}{T}$$

(4) $${\mathrm{Res}}_{1}^{i}=\frac{\mathrm{No}.\text{of times}\;D_{t}^{i}=0\;\text{and}\;D_{t-1}^{i}>0\;}{\mathrm{No}.\text{of times}\;D_{t}^{i}>0\;\text{occurred}}$$

(5) $${\mathrm{Res}}_{2}^{i}=\frac{\int_{t_{0}}^{T}\left(Q_{\text{actual},t}^{i}/Q_{\text{target},t}^{i}\right)dt\;}{T}$$

(6) $${\mathrm{Vul}}^{i}=\frac{\left(\sum_{t=1}^{T}D_{t}^{i}\right)/\mathrm{No}.\text{of times}\;D_{t}^{i}>0\;\text{occurred}}{\sum_{t}Q_{\text{target},t}^{i}}$$

Using the calculated metric results for each water‐using sector, we can further compute the averaged metrics for the entire basin or for specific countries or regions. A general example is given in equation (7):

(7) $$\text{metric}=\sum_{i=1}^{I}\left(\frac{{\text{target}}^{i}}{\sum_{i=1}^{I}{\text{target}}^{i}}\times {\text{metric}}^{i}\right)$$

The sustainability index (SI) is a further metric that combines the three sustainability dimensions for the sectors analyzed (food, energy, and water‐based ecosystem health) and is described in equation (8). *SI*_1_ and *SI*_2_ represent the sustainability calculated by *Res*_1_ and *Res*_2_, respectively:

(8) $$\mathrm{SI}={\left[\mathrm{Rel}\times \mathrm{Res}\times \left(1-\mathrm{Vul}\right)\right]}^{1/3}$$

We acknowledge that these metrics have limitations. For example, equation (8) for sustainability does not consider *excessive resources* such as seasonal flooding as having a positive effect on a wetland. Future studies can develop the sustainability metric approach further by adding a dimensionless index into the calculation. However, a distinction might be needed for species that benefit and those that do not benefit from differing levels of flooding in the wetland. Also, because these are generic metrics, no local stakeholder preferences are considered. To adjust equation (8) to fit the needs of different stakeholders, researchers could conduct interviews, surveys, or workshops to collect data on stakeholder preferences and then apply different weights to *Rel*, *Res,* and *Vul*. Information on stakeholder preferences would also need to capture seasonal variations in the index components, which are currently calculated as an annual average. Seasonal preferences could also be reflected through a weighting approach. In this study, we used an annual time step throughout multiple years, and thus, the results do not account for seasonal, monthly, or daily demands. If a finer time step were used for metric calculations, weights could be placed to favor dry‐season irrigation and dry‐ or wet‐season streamflow conditions, for example.

### 3.2 Coupled Agent‐Based Modeling

A two‐way coupled agent‐based model (ABM‐SWAT) described in Khan et al. (2017) was used in this study to calculate the water availability for irrigated crop production, hydropower generation, and riverine ecosystem health in the Niger River Basin. We defined *agents* as geographical regions with similar hydrological characteristics and administrative structures, as was done in previous applications of agent‐based modeling to water resources management (Giuliani et al., 2014; Yang et al., 2009, 2012). To fully address the development plans for irrigated crop production, hydropower generation, and riverine ecosystem functioning, the Niger River Basin was divided into 13 regions according to the national, political, administrative, and hydrologically relevant boundaries (Khan et al., 2017). Demands for water resources and ecosystem service concerns differ across these regions, and thus, they were identified as autonomous agents. Inside the territory of each agent, the areas were further divided into multiple subbasins (Figure 1) to better reflect spatial parameterization and to evaluate the water availability for irrigated crop production. This coupled model utilizes the SWAT rainfall‐runoff module to simulate available surface water in each subbasin. Individual agents can alter parameters for irrigated crop production and dam modules (crop area and dam operation, respectively) inside the ABM to reflect real‐world adaptive human decisions. The details of the ABM‐SWAT model development, necessary input data, calibration, and validation processes are provided in Khan et al. (2017).

The procedures by which the ABM‐SWAT modeling results were used to calculate the sustainability metrics reflected in equations (1) to (8) are described as follows: First, annual irrigated crop production targets, hydropower generation targets, and targets for riverine ecosystem indicators were imported into the model for individual agents, dams, and identified riverine ecosystem hot spots (broadly used here to refer to areas of exceptional importance for ecosystem services and biodiversity; for a recent review of the concept, see Marchese, 2015), respectively. Second, the ABM‐SWAT model was run for 26 years (1985 to 2010) with a daily time step and with annual two‐way communication between the ABM and SWAT. A pre‐analysis shows that prioritization of water for crop production, hydropower generation, or riverine ecosystem health does not have significant impacts on model results, so we follow Khan et al. (2017) and rank crop production, hydropower generation, and riverine ecosystem as 1, 2, and 3, respectively. Model outputs include annual irrigated crop production, hydropower generation, and the value of the selected riverine ecosystem indicator (7‐day minimum flow) for each agent, dam, and ecosystem hot spot. By applying equations (1) to (6), we then compute the *Rel*, *Vul*, and *Res* metrics for irrigated crop production of each agent, hydropower generation for each dam, and riverine ecosystem health for each ecosystem hot spot. Finally, we average these values to derive basin‐wide, national, or regional measures of *Rel*, *Vul*, and *Res* (equation (7)) and apply equation (8) to compute the sustainability of water availability for food, energy, and the riverine environment.

These methods, including generic metrics of the sustainability and the coupled ABM‐SWAT model, can be easily generalized and applied to any river basin in the world. In this paper, we use the Niger River basin as a demonstration for the feasibility and practicality of our methodology.

## 4 Results

### 4.1 Basin‐Wide Sustainability of Water Availability

#### 4.1.1 Impact of Precipitation Change and Water Infrastructure Development

**Figure 2:**
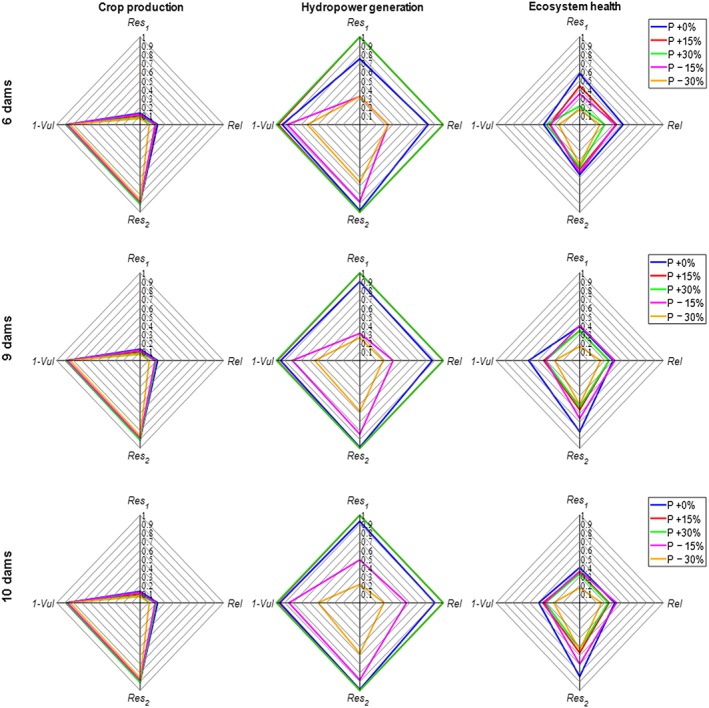
The joint effect of precipitation (*P*) changes and water infrastructure development on basin‐wide water availability reliability (*Rel*), resilience (*Res*_1_ and *Res*_2_), and vulnerability (1*‐Vul*) of irrigated crop production, hydropower generation, and ecosystem health. The blue lines indicate the no‐precipitation‐change condition, and other colored lines represent precipitation increases or decreases. Historical temperature data were used for all these runs.

Using the concept of ex ante scenario analysis, we first tested the effect of precipitation changes combined with dam development while assuming no temperature change. Figure 2 uses radar maps to show the *Rel*, (1*‐Vul*), and *Res* water availability metrics for basin‐wide irrigated crop production, hydropower generation, and riverine ecosystem health. We structured the *y* axis with two different resilience metrics to help readers visualize the sustainability indices, which are represented by the triangular areas. All subsequent radar maps use the same axes.

The ABM‐SWAT model does not simulate rain‐fed crops and flood recession rice production, especially in the Inner Niger Delta, and irrigated crop area is currently relatively small (less than 5% of total crop area in the Niger River Basin). Thus, changes in precipitation levels do not affect basin‐wide irrigated crop production drastically with the exception of the −30% scenario. Under this extreme dry case, *Rel* and *Res*_1_ show an observable decrease in basin‐wide irrigated crop production. In each dam development scenario, the values of *Rel* and *Res*_1_ for irrigated crop production are the lowest among the four sustainability metrics, which means that irrigated crop production has a higher chance of failing to meet annual targets. As irrigated area is set to increase, failures will likely grow in magnitude and impact. In addition, under the different precipitation changes shown in Figure 2, all four metrics of basin‐wide irrigated crop production exhibited little change under alternative water infrastructure development (as can be seen in the same colored lines in the three dam development scenarios). These results indicate that dam development only (without irrigated area expansion) will not significantly increase crop production which is consistent with the conclusion from NBA (2007).

For the whole Niger River Basin, in each dam development scenario basin‐wide hydropower generation shows a larger response to precipitation changes than does basin‐wide crop production. Increasing precipitation (by 15% or 30%) increases basin‐wide hydropower generation moving the *Rel*, *Res*_1_, *Res*_2_, and (1*‐Vul*) closer to 1, indicating *optimal* water sustainability. Decreasing precipitation triggered a drop in all metrics. In each dam development scenario, decreasing precipitation showed an interesting pattern. Under the −15% precipitation scenario, construction of additional dams has the potential to mitigate the effect of decreasing precipitation on *Rel* and *Res*_1_. The effect of Fomi Dam is also substantial, with the dam effectively increasing the *Rel* and *Res*_1_ values. Thus, under the precipitation scenario, the probability of failing to meet an annual basin‐wide hydropower generation target is reduced when more dams are constructed in the basin since the main purpose of these dams is for hydropower generation. However, under the −30% precipitation scenario, dam development effects on *Rel*, *Res*_1_, *Res*_2_, and (1‐*Vul*) are not that straightforward. All metrics under the 9‐dam and 10‐dam scenarios were below those of the 6‐dam scenario. It is certain that more basin‐wide hydropower can be generated under the 9‐dam and 10‐dam scenarios (annual average basin‐wide hydropower generation for 6 dams, 9 dams, and 10 dams is 78,960, 93,358, and 121,872 GWh, respectively), but building more dams does not ensure an improvement in basin‐wide sustainability of water availability for the energy sector. This is because we calculated the basin‐wide metrics based on the averaged value of each individual dam (equation (7)), and the number of dams affects this average value. Our results indicate that under no temperature change and a moderate precipitation decrease (−15%), dam development may be an appropriate policy to maintain hydropower production levels. However, under an extreme precipitation decrease (−30%), a trade‐off occurs between these sustainability metrics for hydropower generation.

The response of the water sustainability metrics for ecosystem health to changes in precipitation was also interesting. Under the 6‐dam scenario, precipitation changes (increases or decreases) negatively affect all four metrics for basin‐wide riverine ecosystem health. This is a result of our selection of the ecologically relevant flow indices: the 7‐day minimum flow and the annual target setting. When precipitation changes, the 7‐day minimum flow may exceed the upper limit or may be below the lower limit of the desired range, which leads to the degradation of riverine ecosystem health. A detailed discussion about the limitations of this indicator is provided in section 5 the discussion section. This suggests that either an increase or a decrease of water in the river will reduce the basin‐wide sustainability of ecosystems, and the decrease might have a worse effect (Figure 2). However, with additional dams constructed in the basin, it is possible to regulate the streamflow with additional storage and potentially decrease the deficit occurrence numbers and improve some aspects of ecosystem sustainability (*Rel*, which represents the probability of missing the target). But modeling results suggest that precipitation changes will still have a negative effect on *Res*_1_, (1*‐Vul*), and *Res*_2_ under future dam development scenarios (Figure 2), except for the *Res*_1_ under −15% precipitation combined in the 9‐dam scenario. It is likely that the regulated streamflow with additional storage alters the streamflow outside of the natural regime which results in the increase of the deficit and the decrease of these metrics. The opposite trend in *Res*_1_ and *Res*_2_ means that the selection of resilience metrics can be critical when we want to use evidence‐based modeling results to inform policy. Further discussion on the difference in resilience metrics is provided in section 5. Interestingly, under different precipitation changes, *Res*_2_ values in the 9‐dam and 10‐dam scenarios are all higher than those in the 6‐dam scenario. This may indicate that with no climate change, constructing additional dams may be beneficial because it would increase the ecosystem deficit recovery rate.

#### 4.1.2 Impact of Temperature Change and Water Infrastructure Development

**Figure 3:**
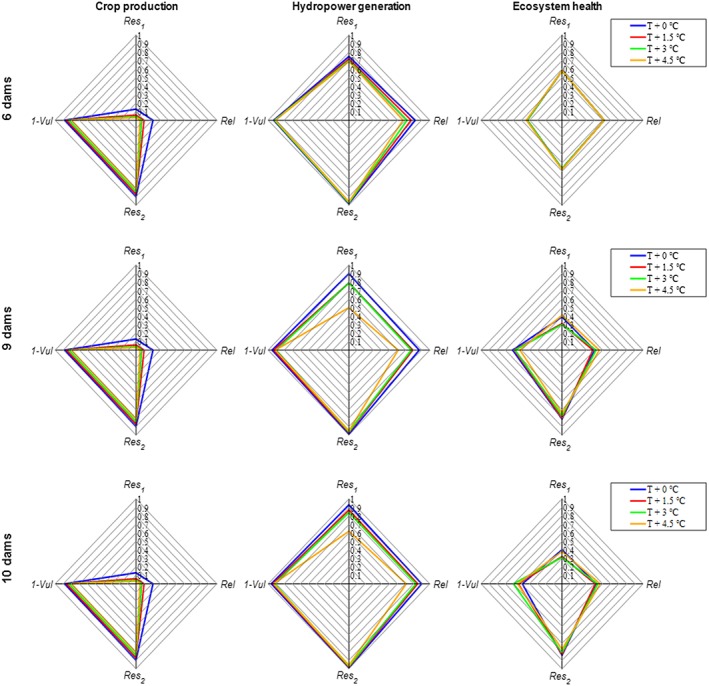
The joint effect of temperature (*T*) changes and water infrastructure development on basin‐wide water availability reliability (*Rel*), resilience (*Res*_1_ and *Res*_2_), and vulnerability (1*‐Vul*) of irrigated crop production, hydropower generation, and ecosystem health. The blue lines indicate the no‐temperature‐change condition, and other colored lines represent temperature increases. Historical precipitation data were used for all these runs.

Figure 3 shows the effect of temperature change combined with dam development assuming no precipitation change. As temperature is a key factor in the simulation of irrigated crop production in SWAT, we see a detectable negative impact on basin‐wide water sustainability metrics for irrigated crop production under increasing temperature conditions across all three dam development scenarios. Each crop has a suitable temperature range, and crop growth declines to zero when temperature limits have been reached (Neitsch et al., 2011). In addition, dam development has nearly no implications for irrigated crop production under different temperature changes due to the dams' main purpose for hydropower generation.

All temperature increases have a negative effect on hydropower generation, and the negative effect increases with the number of dams because of the greater dam surface area evaporation and the averaged value of each dam (equation (7)). It is noteworthy that when temperature remains unchanged or increases by 1.5 °C, all four metrics of basin‐wide hydropower generation are higher in the 9‐dam or 10‐dam scenarios than they are in the 6‐dam scenarios. Under the +3 and +4.5 °C temperature scenarios, changes in the four metrics are not consistent. For the 10‐dam scenario under extreme dry conditions (+4.5 °C), only *Res*_1_ decreases. These findings indicate that under a slight temperature change (+1.5 °C) the construction of additional dams improves the sustainability of hydropower production. Under moderate and extreme temperature changes (+3 or +4.5 °C), there are trade‐offs across the four metrics. With the Fomi Dam in place, hydropower generation under the most extreme temperature increase can be maintained close to the level of no temperature increase and no dam development. Since Fomi had the second‐largest full storage among the 10 dams and is the most upstream planned large dam, it can significantly regulate the downstream flows. Streamflow will decrease under the extreme temperature increase condition (due to a higher evapotranspiration rate), but the additional storage in Fomi can compensate this negative effect and maintain a similar level of hydropower generation at the basin scale compared to the baseline. Basically, these results confirm that dam development contributes to the sustainability of water availability for hydropower generation.

The sustainability of water availability for ecosystem health does not appear to be significantly affected by temperature changes based on the ecologically relevant flow indices we used in this study because they are not temperature sensitive. Under future dam development scenarios, temperature increases led to opposite trends of *Rel*, *Res*_1_, and (1*‐Vul*). *Res*_2_ always decreased with increasing temperature. Particularly, (1*‐Vul*) and *Res*_2_ values under the 9‐dam and 10‐dam scenarios for each temperature level are higher than the values under the 6‐dam scenario, while the other two metrics (*Rel* and *Res*_1_) are lower. Dam development with additional storage is likely to mitigate the negative impact of temperature increases on ecosystem deficit severity and recovery rate.

#### 4.1.3 Joint Impact of Climate Change and Water Infrastructure Development

**Figure 4:**
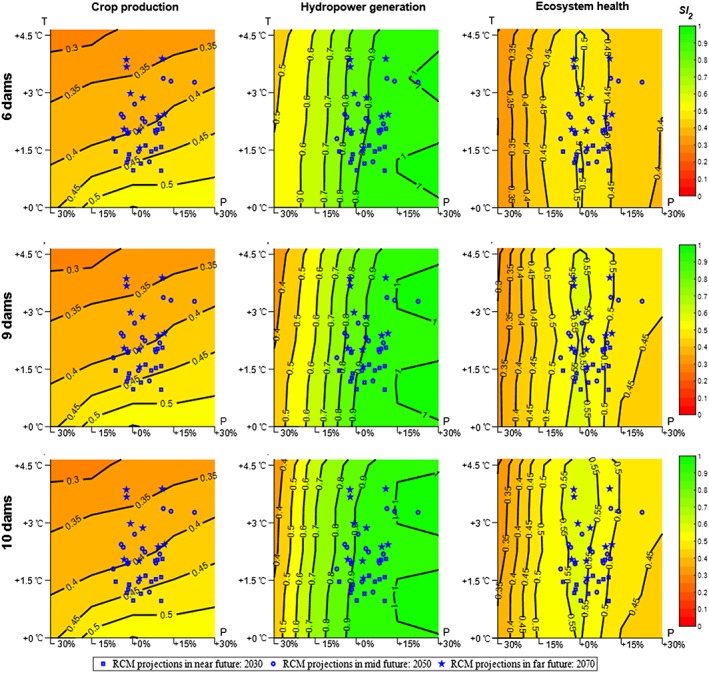
Climate stress test by resilience (*Res*_2_) on basin‐wide water availability sustainability index of irrigated crop production, hydropower generation, and ecosystem health under different water infrastructure development conditions. The blue dots represent regional climate model (RCM) projections for different periods (2030, 2050, and 2070) to inform the likelihood of future climate conditions.

Using the findings described in the previous sections, we conducted a climate stress test (Brown et al., 2012) on the sustainability of water availability for crop production, hydropower generation, and ecosystem health and evaluated the effect on dam development at the same time. We used only *Res*_2_ for the sustainability calculation (*SI*_2_); a demonstration and results based on *Res*_1_ are given in the supporting information (Figure S1). Figure 4 shows these results, with red and green indicating lower and higher sustainability index values, respectively. We used future climate projections from seven regional climate models run under representative concentration pathways 4.5 and 8.5 to visualize the likelihood of future climate change. Descriptions of these seven regional climate models are provided in the supporting information (Table S1). The outputs of these regional climate models were obtained from the Coordinated Regional Climate Downscaling Experiment team, n.d. (http://www.cordex.org/) and were downscaled using quantile matching (for precipitation and temperature) and K‐nearest neighbor search (for humidity and wind). A complete description of the downscaling algorithms can be found in Angelina et al. (2015). The downscaled temperature and precipitation changes for the Niger River Basin are superimposed in Figure 4 as blue dots. We also indicated *near*, *mid*, and *far* future (2030, 2050, and 2070) as different symbols in Figure 4.

Figure 4 lends support to the idea that temperature is the dominant driver of changes in irrigated crop production (horizontal pattern of stress test results), and precipitation is the dominant driver of changes in hydropower generation and ecosystem health (vertical pattern of stress test results). The likely future climate domain (where the blue dots are located) will result in water availability sustainability index values between 0.33 and 0.48 for crop production across all three dam development scenarios, with a baseline value of 0.55. Dam development cannot mitigate the negative impact of temperature increases on irrigated crop production. The likely future climate domain will result in water availability sustainability index values ranging from 0.76 to 1.00 for hydropower generation in the 6‐dam scenario with a baseline value of 0.91. Dam development does help to mitigate climate change impact, while the water availability sustainability index value for the 9‐dam scenario lies between 0.76 and 1.00 and that of the 10‐dam scenario lies between 0.82 and 1.00. The sustainability index values for ecosystem health in the 6‐dam scenario, the 9‐dam scenario, and the 10‐dam scenario range from 0.42 to over 0.5, 0.47 to over 0.55, and 0.47 to over 0.55, respectively. Dam development can potentially mitigate adverse climate change impacts for some aspects of ecosystem health. Similar to what have shown in sections 4.1.1 and 4.1.2, the results of previous analyses suggest that under likely future climate changes, dam development can increase the sustainability of water availability for basin‐wide hydropower generation and ecosystem health but not for irrigated crop production.

### 4.2 National and Regional Sustainability of Water Availability

#### 4.2.1 Water Availability Sustainability

Results in section 4.1 provide an overall picture of basin‐wide sustainability of water availability but do not address the inconsistent development and heterogeneity among different regions or countries. For a transboundary river basin like the Niger, it is beneficial for policy makers to understand changes in regional and national sustainability under different external drivers. Considering demographic, ecological, fishery distribution, country area, water quantity, and socioeconomic factors, we identified five countries along the main stem of the Niger: Guinea, Mali, Niger, Nigeria, and Cameroon and three regions of special interest: Office du Niger (major irrigated crop production), Inner Niger Delta (key ecosystem hot spot), and Outlet Niger Delta (key ecosystem hot spot) for regional‐level analyses (e.g., Bhattacharyya et al., 2015; Kuper et al., 2003; Liersch et al., 2013; NBA, 2007; Ogilvie et al., 2010; Passchier et al., 2005; Sidibé & Williams, 2016; Zwarts et al., 2006). The relative location of these countries and locations from upstream to downstream are as follows: Guinea, Mali, Office du Niger, Inner Niger Delta, Niger, Nigeria, Outlet Niger Delta, and Cameroon (the Benue River). We highlight the *Res*_2_ changes of crop production, hydropower generation, and ecosystem health in these areas as demonstrations. Results for *Rel*, *Res*_1_, (1*‐Vul*), *SI*_1_, and *SI*_2_ are provided in the supporting information.

**Figure 5:**
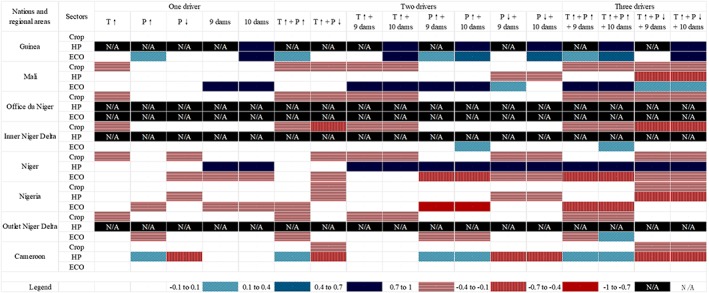
The joint effect of climate change and water infrastructure development on the maximum change of national and regional resilience (*Res*_2_) of crop production, hydropower generation, and ecosystem health compared to the baseline scenario. Maximum index value changes between +0.1 and −0.1 are defined as *no change* (colored in white). Index value changes from 0.1 to 0.4, from 0.4 to 0.7, and above 0.7 are defined as *minor*, *moderate*, and *major* changes, respectively. Blue colors (from light to dark) are used to represent positive changes, and red colors (from light to dark) are used to represent negative changes. Abbreviations: *T* = temperature; *P* = precipitation; crop = crop production; HP = hydropower generation; ECO = ecosystem health.

Figure 5 is a heatmap that shows the joint effect of climate change and water infrastructure development on the maximum changes of national and regional *Res*_2_ metrics compared to the baseline scenario for all three water‐using sectors. Changes of between +0.1 and −0.1 in metric values are defined as *no change* (colored in white). Changes ranging from 0.1 to 0.4, from 0.4 to 0.7, and above 0.7 are defined as *minor*, *moderate*, and *major* changes, respectively. Blue colors (from light to dark) are used to represent positive changes, and red colors (from light to dark) are used to represent negative changes.

As Figure 5 shows, most changes are negative, except for some hydropower generation and ecosystem health outcomes under the dam development scenarios. This indicates that, under the combined impact of future climate change and dam development, *Res*_2_ for irrigated crop production, hydropower generation, and ecosystem health will mostly decline compared to that in the baseline scenario. For example, if we only look at the impact of a temperature increase (*T*↑) or precipitation decline (*P*↓), *Res*_2_ values decrease for the three sectors in all national and regional sample cases. No countries or regions will benefit when temperatures increase or precipitation decreases. Some aspects of ecosystem health in Guinea and Cameroon will benefit from precipitation increase. Some aspects of ecosystem health in Mali and Niger could benefit from dam development if releases are used to reduce the risk of 7‐day minimum flows.

When we consider two or three drivers together, no clear trend can be identified because positive and negative effects from a single driver may be canceled out or strengthened. The combination of the increased temperature and increased precipitation (*T*↑ + *P*↑ column); the increased temperature, increased precipitation, and 9 dams (*T*↑ + *P*↑ + 9‐dam column); and the increased temperature, increased precipitation, and 10 dams (T↑ + P↑ + 10‐dam column) suggests that the construction of additional dams will increase *Res*_2_ values for hydropower generation in Guinea and Niger. Meanwhile, the *Res*_2_ values for ecosystem health in Guinea, Mali, Inner Niger Delta, and Outlet Niger Delta might also potentially increase under these conditions. However, the construction of additional dams may have a negative effect on ecosystem health in Niger and Nigeria. The combination of (*T*↑ + *P*↓ column), (*T*↑ + *P*↓ + 9‐dam column), and (*T*↑ + *P*↓ + 10‐dam column) suggests that the construction of additional dams can contribute to an increase in *Res*_2_ values for hydropower generation in Guinea and Niger, as well as an increase in aspects of ecosystem health in Guinea and Mali. However, hydropower generation in downstream Mali and Nigeria would be adversely affected.

In general, these results suggest that construction of additional dams will not significantly affect the crop production sustainability (NBA, 2007) by previous analyses in section 4.1. Water infrastructure development does have the potential to mitigate negative climate change impacts on hydropower generation and ecosystem health in parts of the basin, but the effect is certainly not basin wide. For example, under the 9‐dam and 10‐dam scenarios, the *Res*_2_ values for ecosystem health in Niger and Nigeria will decrease 10–40%. Since all new dams are planned to be located upstream of these two countries, it is plausible that these new upstream dams alter the flow regime so that the negative effect on 7‐day minimum flows accumulates by the time flows reach Niger and Nigeria. More detailed analysis is needed when additional data (such as real reservoir operational rules for these dams) become available.

We compare our results with previous NBA studies to make sure that our modeling outcomes are consist with NBA's description. NBA (2007) concludes that dam development will significantly enhance hydropower generation but will not have a significant negative effect on crop production since most crop production in the Niger Basin is rain fed which matches our modeling results. For ecosystem results, different papers draw different conclusions, among others, due to differing models, data, and assumptions. The most popular debate is focused on the influence of the Fomi Dam on the Inner Niger Delta; while some studies remain neutral about potentially negative effects (Passchier et al., 2005), others suggest that Fomi will alter the flow regime in the Inner Niger Delta in the dry and flood seasons (Ghile et al., 2014; Kuper et al., 2003; NBA, 2007). It is difficult to compare our ecosystem results with these papers. Given our selection of the 7‐day minimum flow indicator and annual analyses, sustainability analysis for ecosystem health in this paper should be interpreted with care (see also section 5).

We summarize some interesting patterns here for the policy analysis and discuss some entry points for reducing negative and improving positive interactions across the Nexus sectors and geographies. First, these results suggest that Guinea will support all dam development since it will be a beneficiary of both hydropower and ecosystem sectors under all climate conditions. Second, irrigated crop production in Mali, Niger, and Nigeria will most likely be negatively affected by future climate, and since dam development is not capable of mitigating this effect, these countries can join forces to develop alternative mitigation and adaptation strategies to further ensure the resilience of food production. Third, basin‐wide dam development will cause an internal conflict inside Niger's hydropower sector and ecosystem health and the future precipitation change might worsen this conflict. Therefore, within the country debate on dam development is needed in addition to ongoing debates around Fomi dam. Finally, as the most downstream country in the basin, our modeling results suggest that all sectors in Nigeria will be negatively impacted under these scenarios (i.e., cells are either white or red in Figure 5). However, they have the highest GDP per capita in the region ($2,177 vs. Niger: $363; Guinea: $508; Mali: $780) according to the World Bank Database 2016; it is possible that Nigeria can develop some compensation mechanism for these upstream countries to support alternative Niger basin development paths.

Given a transboundary river basin like the Niger, it is likely that policies in the different countries will vary based on national interests (e.g., national security and domestic demand for food and energy) with less consideration about the potential impact on other countries. We hope that the modeling implemented here can support discussions at the level of the NBA, which combines transboundary interests of the nine basin countries. Our results show that there are incentives for riparian states to cooperate with others to utilize the mutual resources and to obtain the mutual benefits (Bekchanov et al., 2015). In addition, nexus‐related conflicts inside each riparian country will increase complexity. Country governments should also coordinate internally for the development of different sectors and balance the trade‐offs to obtain the co‐benefits (Rasul, 2014). By cooperation among different countries and coordination among different sectors, the mutual resources can be better utilized, and the nexus development in transboundary rivers will be more sustainable.

#### 4.2.2 Economic Performance

As different metrics are designed to reflect different aspects of sustainability of water availability, trade‐off analysis among different countries provides another layer of information for policy analysis. As irrigated crop production and hydropower generation are directly related to national GDP, we will further explore these two water‐using sectors in economic trade‐off analyses. While it is understood that ecosystem health is fundamental to national GDP and human health, it is beyond the scope of this paper to assess ecosystem health in economic trade‐off analysis. Instead, we assess whether construction of additional dams contributes to crop and energy production in different scenarios. Here we focus on annual average irrigated crop production and hydropower generation under three climate scenarios as examples: the baseline scenario (*T* +0 °C, *P* +0%), the driest scenario (*T* +4.5 °C, *P* −30%), and the wettest scenario (*T* +0 °C, *P* +30%). Results under 17 additional climate scenarios are presented in the supporting information (Tables S2 and S3).

**Figure 6:**
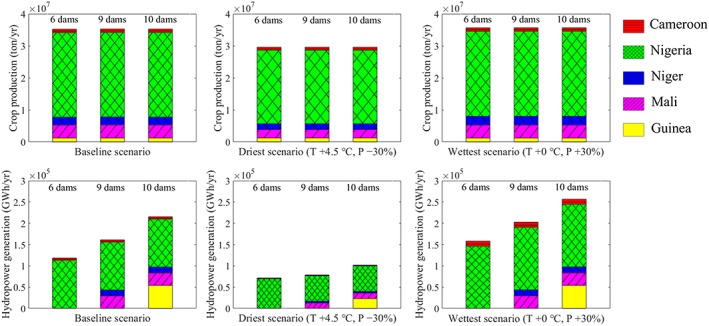
The joint effect of climate change and water infrastructure development on basin‐wide and national annual irrigated crop production and hydropower generation in the baseline, driest, and wettest scenarios. Abbreviations: *T* = temperature; *P* = precipitation.

Figure 6 shows the differences in annual average irrigated crop production and hydropower generation at the basin level. Results indicate that basin‐wide irrigated crop production will decrease (by approximately 18%) under the driest scenario but will increase slightly under the wettest scenario. As has been discussed before, temperature is the main driver of changes in irrigated crop production. That is why under the wettest scenarios (only precipitation changes), crop production changes are small. However, increasing temperature may exceed the suitable temperature range, reducing irrigated crop production in the driest scenario. Dam development alone does not have significant effects on irrigated crop production, and the ratio of country level to basin‐wide crop production is not affected by climate change or dam development.

As the dominant driver of changes in hydropower generation is changes in precipitation (section 4.1.3), changes between the driest and the wettest scenarios are obvious. In the wettest scenario, increased precipitation supports increased hydropower generation. On the other hand, in the driest scenario, streamflows decrease negatively affecting hydropower generation. Also, as described in section 4.1.2, temperature also negatively influences hydropower generation. Thus, under the driest scenario, the combination of decreased precipitation and increased temperature will dramatically reduce hydropower generation. From a country‐level perspective, hydropower generation in Guinea, Mali, and Niger benefit from dam development under extreme climate conditions. For Cameroon, hydropower generation remains unchanged with dam development. For Nigeria, in the baseline and wettest scenarios hydropower generation remains unchanged under dam development; however, in the driest scenario, hydropower generation decreases following dam development. Note that water infrastructure development is not capable to compensate for the negative effect under the driest scenario since new water infrastructure will not increase the physical water availability at the basin scale (using annual time steps). Therefore, all countries in the basin, with or without new dams, will suffer from reduced hydropower generation. From a basin‐wide perspective, in all selected climate scenarios, the basin will achieve maximum hydropower generation with the construction of ten dams including the Fomi.

## 5 Discussion

### 5.1 Selection and Calculation of Sustainability Metrics

When we use generic metrics to represent the sustainability of water availability for a complex socioecological system, understanding their true meaning, their assumptions, and the ways in which they are calculated is critical for meaningful policy support. Key elements that need to be further developed include the time step used for metric calculation and the selection of different resilience metrics.

As the study combines water use preferences spanning annual ecosystem health, seasonal irrigated crop production, and daily hydropower generation, all values are assessed at an annual basis only. If sufficient data are available, hydropower generation and hydroecologic indicators can be disaggregated into daily targets with indicator values changing across seasons and days. Table 2 compares annual and daily metric calculations used in this study under no climate change and dam development for basin‐wide hydropower generation and riverine ecosystem health. Metrics calculated at a daily time step always have lower values than do annual results. This might be caused by the spatial, temporal, and interannual variation of meteorological and hydrological characteristics in this basin (Khan et al., 2017). Analysis on an annual time scale ignores variations in daily streamflow and thus fails to record daily deficits in water availability. In addition, an annual time scale might miss intraannual floods and droughts. If daily targets need to be met, the number of deficits grows. An appropriate time scale should be chosen for metrics calculations with consideration given to the management purpose and time horizon of the analysis.

**Table 2:** Effect of Computational Time Step (Annual or Daily) on Reliability, Vulnerability, Resilience, and Sustainability Metrics of Water Availability for Hydropower Generation and Ecosystem Health

Metrics	Hydropower		Ecosystem	
	Annual	Daily	Annual	Daily
Reliability	0.82	0.65	0.52	0.14
Resilience 1	0.75	0.30	0.58	0.01
Resilience 2	0.98	0.81	0.57	0.46
1‐vulnerability	0.93	0.61	0.43	0.18
Sustainability 1	0.83	0.49	0.51	0.06
Sustainability 2	0.91	0.68	0.50	0.23

The two resilience metrics used in this study represent different aspects of the resilience concept. *Res*_1_ is the probability that a system will recover to its original state after an external shock, while *Res*_2_ is the “system functionality” recovery path (against time) after an external shock. The calculation for *Res*_1,_ which is a statistic calculation of whether deficit occurs or not, is simpler than the calculation of *Res*_2_, which is integrated over the entire time horizon. Table 2 provides a comparison of sample results of different resilience metrics. The differences between resilience metrics calculated at an annual versus a daily time step are always larger in *Res*_1_ than they are in *Res*_2_. We find that the resilience of hydropower systems (or ecosystems) in the Niger River Basin is at an acceptable range (0.75 for hydropower and 0.58 for ecosystem health) if we use an annual scale *Res*_1_, but resilience is low (0.30 for hydropower and 0.01 for ecosystem health) if we use a daily scale *Res*_1_. However, this situation is not likely to happen if we use *Res*_2_, because the annual and daily scale values are much closer for *Res*_2_ than they are for *Res*_1_. This means that *Res*_2_ is probably a more robust metric if we want to evaluate the resilience of a complex system.

### 5.2 Limitations

In this study, target settings are based on historical long‐term average data. Setting forward‐looking targets based on projected population growth, rather than past or current production levels, would affect the sustainability metrics. In addition, only irrigated crop production is considered in this study, while most food is produced with rain‐fed production, and livestock production and fisheries are both of high importance and depend on adequate water availability. Hence, this is a relatively simplified food production model.

Furthermore, we look at ecosystem health by assessing whether the 7‐day minimum flow target is met at the nodes in the model that are identified as fishery hot spots. This is a computationally easy indicator that can be relatively easily included in the model. However, ecosystems are obviously very complex systems made up of an intricate mixture of microbiological and physicochemical processes and containing an enormous diversity of flora and fauna. The health of such aquatic ecosystems is normally dependent on many factors, such as water quality and composition, and is based on hydraulic regimes with alternations of low‐ and high‐discharge flows. Changes in infrastructure development affect elements of ecosystem health differentially. While some species thrive on flood pulse systems with higher flows followed by reduced flows, others thrive better on larger flows and floodplains, as do fisheries. An ideal ecosystem health indicator would be composed of several subindicators reflecting the various relationships. The 7‐day minimum flow indicator that we used here represents only one part of the required flood dynamics needed to sustain such ecosystems, and misses many other aspects, such as flow connectivity, that might be affected by dam development. One notable modeling result is that when we only use this indicator to reflect ecosystem health, dam development might be favored as reservoirs tend to increase low flows. However, the consequent elimination of larger (flushing) flows, reduced inundation of floodplains (where fish spawn and fodder and rice grow), effects on water temperature, and barriers that dams represent for fish migration are currently ignored in our analysis. Hence, this single indicator is just a starting point and far from enough to completely quantify riverine ecosystem health. Therefore, the findings of this study on ecosystem health should be interpreted with care, especially considering how important aquatic ecosystems in the Niger Basin are for millions of poor people whose livelihood and even survival depend on it.

### 5.3 Potential Future Research

In this study, the sustainability of the nexus is assessed from the perspective of water availability. This measure is limiting in its ability to represent the sustainability of the overall nexus. Enriching the sustainability of the overall nexus rather than just focusing on unilateral sustainability of water availability requires a more comprehensive model which fully couples the food and energy sectors. Overall, this paper can be viewed as a step toward achieving this goal. Several future research directions can be explored in water‐food‐energy‐ecosystem nexus analyses. To better promote the development of the nexus, interactions and feedbacks among different sectors must be further addressed. Thus, future research directions should include (1) coupling water‐food‐energy models with multiple environmental indicators (some possible tools such as the lifecycle analysis, complexity theory, sustainable supply [value] chains, and system dynamics are described in Bazilian et al., 2011; Cai et al., 2018; Halbe et al., 2015) and (2) adding more elements (such as multiple water resources, different energy sources, food production‐related factors, and social and economic factors) into the coupled water‐food‐energy‐ecosystem model. Other future research can address different types of agricultural production and incorporate multiple ecological indicators to better reflect riverine water demand for various purposes.

## 6 Conclusion

Meeting the growing demands for fresh water, food, and energy while maintaining the sustainability of ecosystems is a pressing global challenge. This paper used the Niger River Basin in West Africa to apply a newly developed combined agent‐based hydrological modeling framework for assessing the impacts of climate change and socioeconomic development on the sustainability of water availability in a water‐food‐energy‐ecosystem nexus. Trade‐offs among different countries and regions were also examined for better policy assessment. A set of generic metrics that includes reliability, resilience, and vulnerability was applied to evaluate the sustainability of water availability for irrigated crop production, hydropower generation, and ecosystem health simultaneously in the Niger River Basin. These metrics are based on the deficit concept, which we defined as any gap between target and actual production or between resource demand and supply. The SWAT model simulates the natural hydrologic cycle, and the ABM evaluates how human behavior influences this cycle; thus, the ABM‐SWAT model represents the interactions between human behavior and natural processes. In total, 60 scenarios are tested using this model, which represent a wide range of future climate change conditions (precipitation and temperature) and potential socioeconomic conditions (water infrastructure development).

Modeling results suggest that the sustainability of water availability for irrigated crop production is sensitive to temperature, while the sustainability of hydropower generation and ecosystem health is mostly sensitive to precipitation and dam development. Under likely future climate change, the sustainability metric *Res*_2_ shows that dam development can increase the sustainability of water availability for basin‐wide hydropower generation and ecosystem health to some extent, while irrigated crop production basically remains unaffected because the irrigated area accounts for less than 5% of total the crop area in the Niger River Basin. From a national and regional water sustainability perspective, under climate change dam development has the potential to facilitate hydropower generation in Guinea and Niger, as well as ecosystem health in Guinea and Mali, subject to the methodological limitations stated in the manuscript. From an actual economic performance perspective, excluding ecosystem health from the economic analysis, dam development has no significant effect on current low levels of irrigated crop production but can mitigate negative climate change impact for hydropower generation.

The selection of metrics for resilience and the time step used to calculate these metrics could be critical for the results, as the sustainability of water availability varies based on the metrics used. Suggested research directions for follow‐up studies using the ABM‐SWAT model could include a focus on specific crop types, expanded quantification of ecosystem health, deeper analysis of national and regional level results, overall assessment of nexus or individual sectors rather than just water availability, and expansion of irrigated area and hydropower generation in tandem with reservoir development.

## Supporting information

Figure S1

Table S1
