# Supplementary material for: Quantifying the Sustainability of Water Availability for the Water‐Food‐Energy‐Ecosystem Nexus in the Niger River Basin
Source: Earths Future. 2018 Sep 22;6(9):1292–310. doi: 10.1029/2018EF000923 (PMC6473704; doi:10.1029/2018EF000923)
Supplement: Supplementary file 1 — Figure S1 [file EFT2-6-1292-s001.pdf]

Earth's Future

Supporting Information for

Quantifying the Sustainability of Water Availability for the Water-Food-Energy-Ecosystem Nexus in the Niger River Basin

Jie Yang[1,2], Y. C. Ethan Yang[2*], Hassaan F. Khan[3], Hua Xie[4], Claudia Ringler[4], Andrew Ogilvie[5], Ousmane Seidou[6,7], Abdouramane Gado Djibo[8], Frank van Weert[9], and Rebecca Tharme[10]

[1.] State Key Laboratory of Eco-hydraulics in Northwest Arid Region of China (Xi'an University of Technology), Xi'an, 710048
[2.] Department of Civil and Environmental Engineering, Lehigh University, Bethlehem, PA 18015, USA
[3.] Department of Civil and Environmental Engineering, University of Massachusetts, Amherst, MA 01003, USA
[4.] International Food Policy Research Institute, Washington, DC, 20006, USA
[5.] IRD UMR G-EAU, BP 5095, 34196 Montpellier CEDEX 5, France
[6.] Department of Civil Engineering, University of Ottawa, Ottawa, ON, Canada
[7.] United Nations University, Institute for Water, Environment and Health, Hamilton, ON, Canada
[8.] Wetlands International, Bamako, Mali
[9.] Wetlands International, 6700 AL Wageningen, The Netherlands
[10.] Riverfutures, Cressbrook, Derbyshire, SK17 8SX, UK
[*] Corresponding author, +1-610-758-5685; yey217@lehigh.edu

Contents of this file

Figures S1 to S6

Additional Supporting Information (Files uploaded separately)

None

Introduction

The supplement provide additional figures for the manuscript.

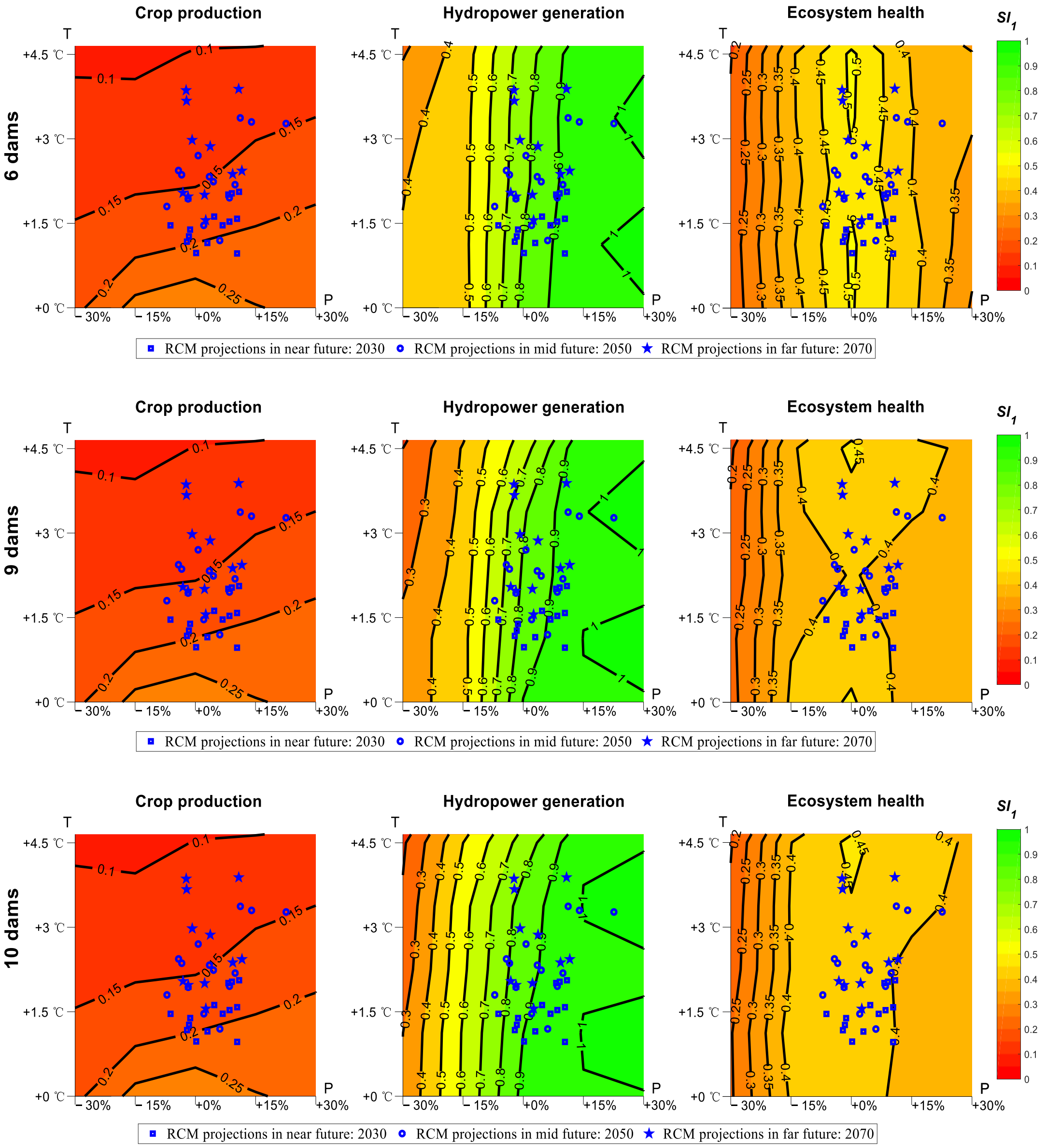

Figure S1. Climate stress test by resilience (***Res₁***) on basin-wide water availability sustainability index of irrigated crop production, hydropower generation, and ecosystem health under different water infrastructure development conditions. The blue dots represent regional climate model (RCM) projections for different periods (2030, 2050, and 2070) to inform the likelihood of future climate conditions

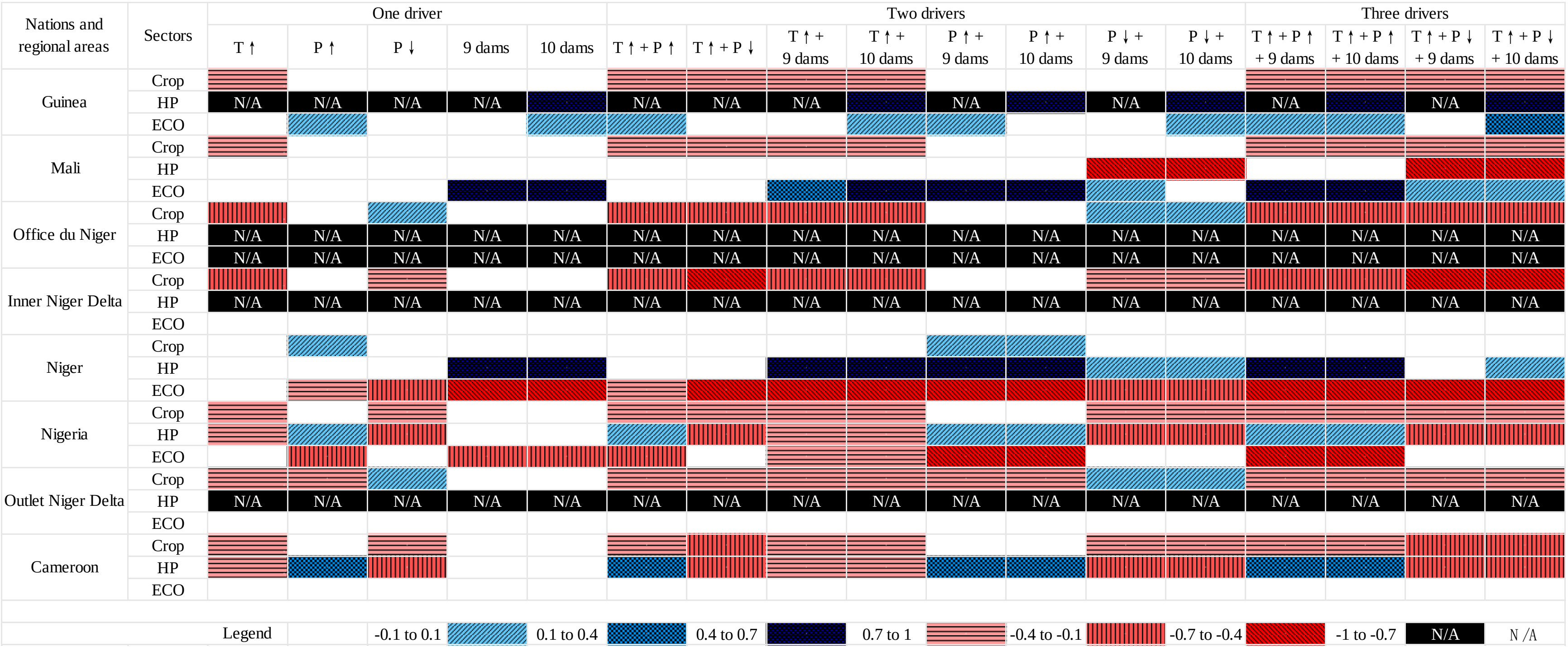

Figure S2. The joint effect of climate change and water infrastructure development on the maximum change of national and regional resilience (***Rel***) of crop production, hydropower generation, and ecosystem health compared to the baseline scenario. Maximum index value changes between +0.1 and −0.1 are defined as "no change" (colored in white). Index value changes from 0.1 to 0.4, from 0.4 to 0.7, and above 0.7 are defined as "minor," "moderate," and "major" changes, respectively. Blue colors (from light to dark) are used to represent positive changes and red colors (from light to dark) are used to represent negative changes. Abbreviations: T, temperature; P, precipitation; crop, crop production; HP, hydropower generation; ECO, ecosystem health

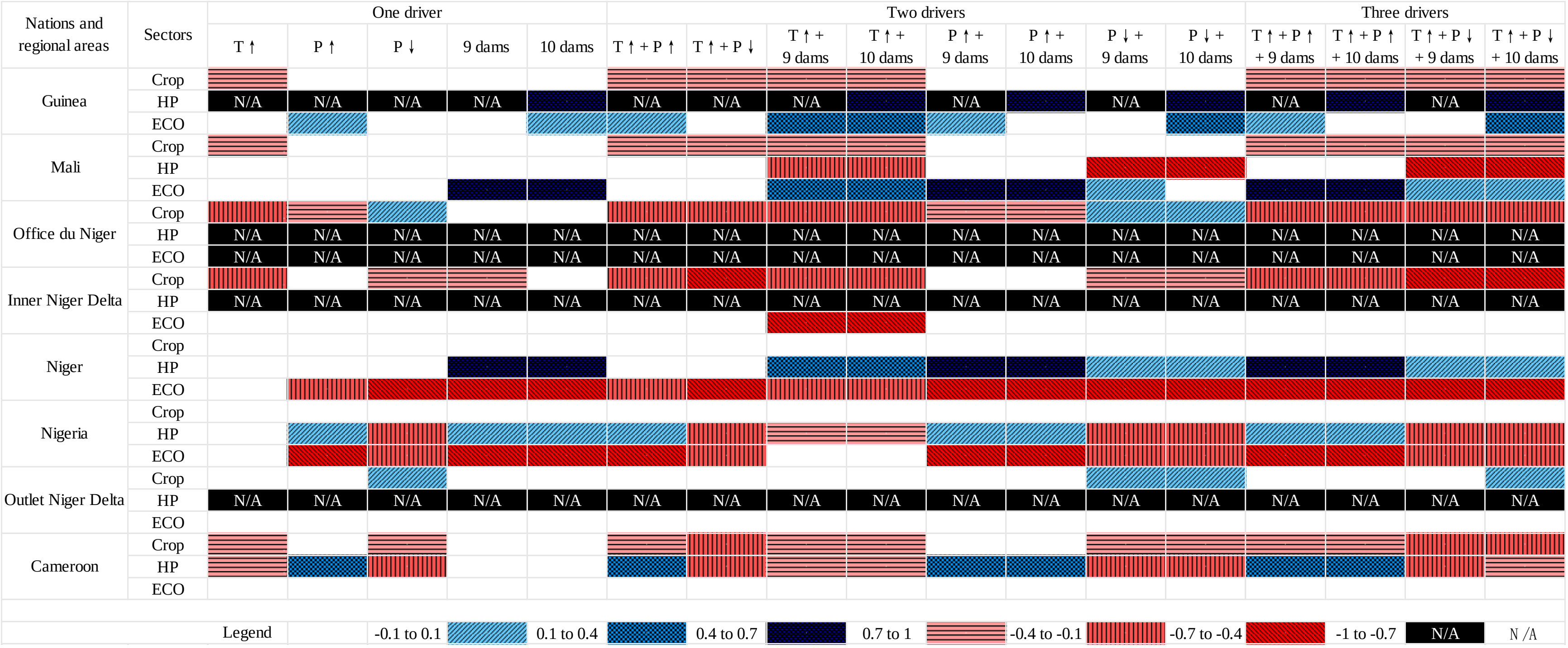

Figure S3. The joint effect of climate change and water infrastructure development on the maximum change of national and regional resilience (***Res*₁**) of crop production, hydropower generation, and ecosystem health compared to the baseline scenario. Maximum index value changes between +0.1 and −0.1 are defined as “no change” (colored in white). Index value changes from 0.1 to 0.4, from 0.4 to 0.7, and above 0.7 are defined as “minor,” “moderate,” and “major” changes, respectively. Blue colors (from light to dark) are used to represent positive changes and red colors (from light to dark) are used to represent negative changes. Abbreviations: T, temperature; P, precipitation; crop, crop production; HP, hydropower generation; ECO, ecosystem health

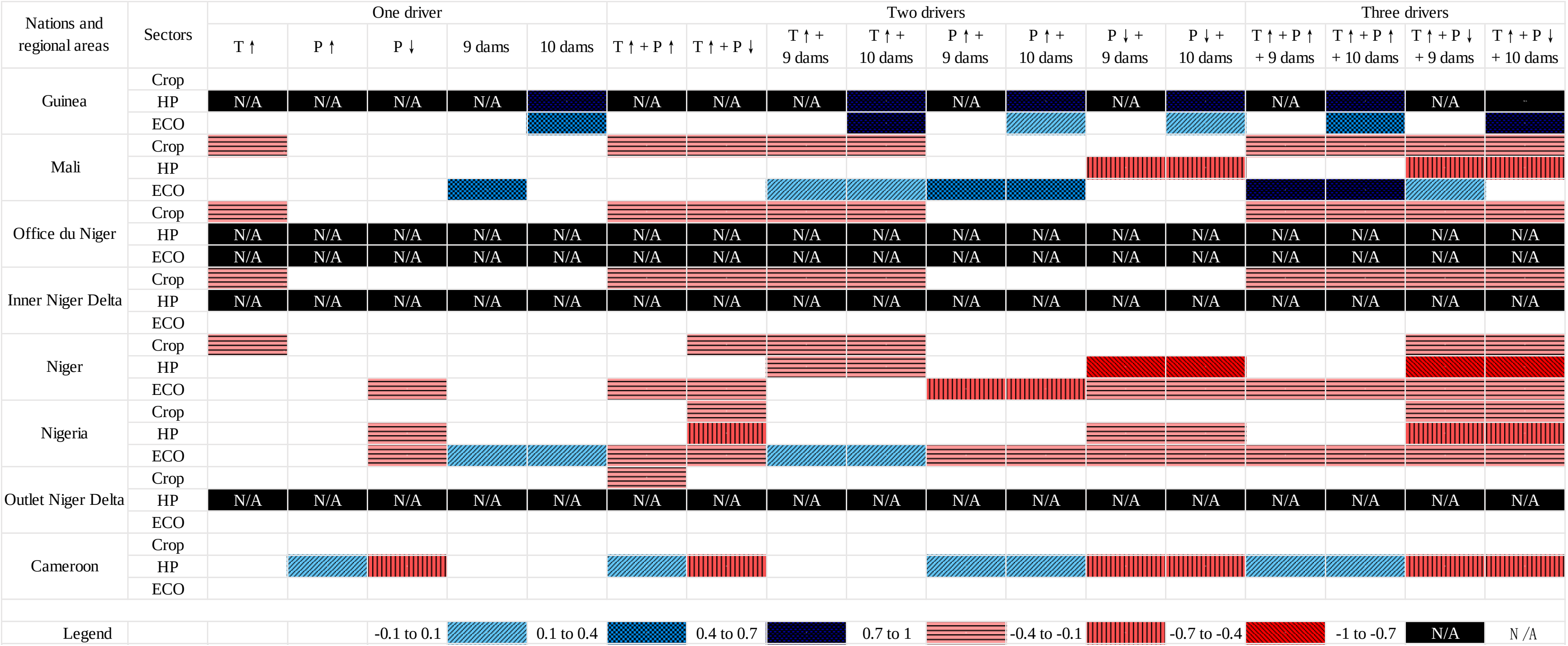

Figure S4. The joint effect of climate change and water infrastructure development on the maximum change of national and regional resilience (***1-Vul***) of crop production, hydropower generation, and ecosystem health compared to the baseline scenario. Maximum index value changes between +0.1 and −0.1 are defined as “no change” (colored in white). Index value changes from 0.1 to 0.4, from 0.4 to 0.7, and above 0.7 are defined as “minor,” “moderate,” and “major” changes, respectively. Blue colors (from light to dark) are used to represent positive changes and red colors (from light to dark) are used to represent negative changes. Abbreviations: T, temperature; P, precipitation; crop, crop production; HP, hydropower generation; ECO, ecosystem health

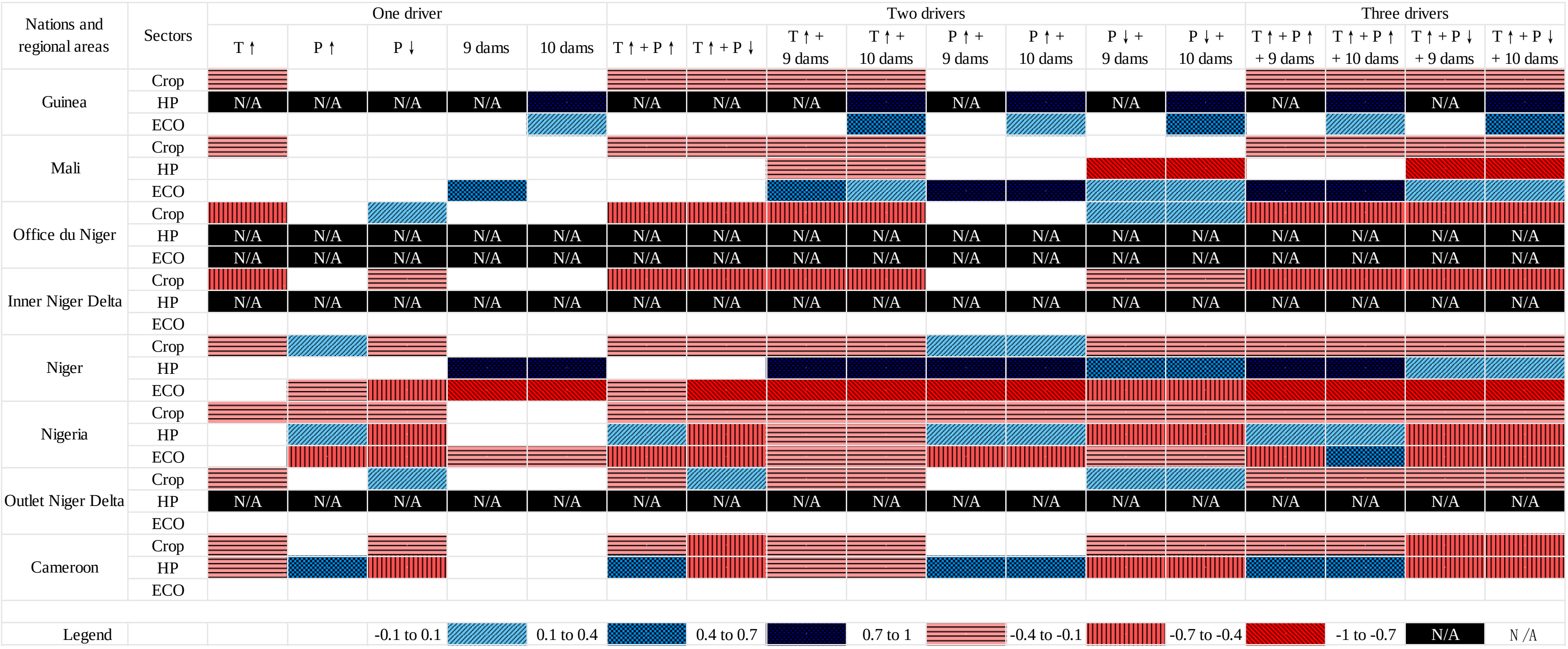

Figure S5. The joint effect of climate change and water infrastructure development on the maximum change of national and regional resilience (***SI**₁*) of crop production, hydropower generation, and ecosystem health compared to the baseline scenario. Maximum index value changes between +0.1 and −0.1 are defined as "no change" (colored in white). Index value changes from 0.1 to 0.4, from 0.4 to 0.7, and above 0.7 are defined as "minor," "moderate," and "major" changes, respectively. Blue colors (from light to dark) are used to represent positive changes and red colors (from light to dark) are used to represent negative changes. Abbreviations: T, temperature; P, precipitation; crop, crop production; HP, hydropower generation; ECO, ecosystem health

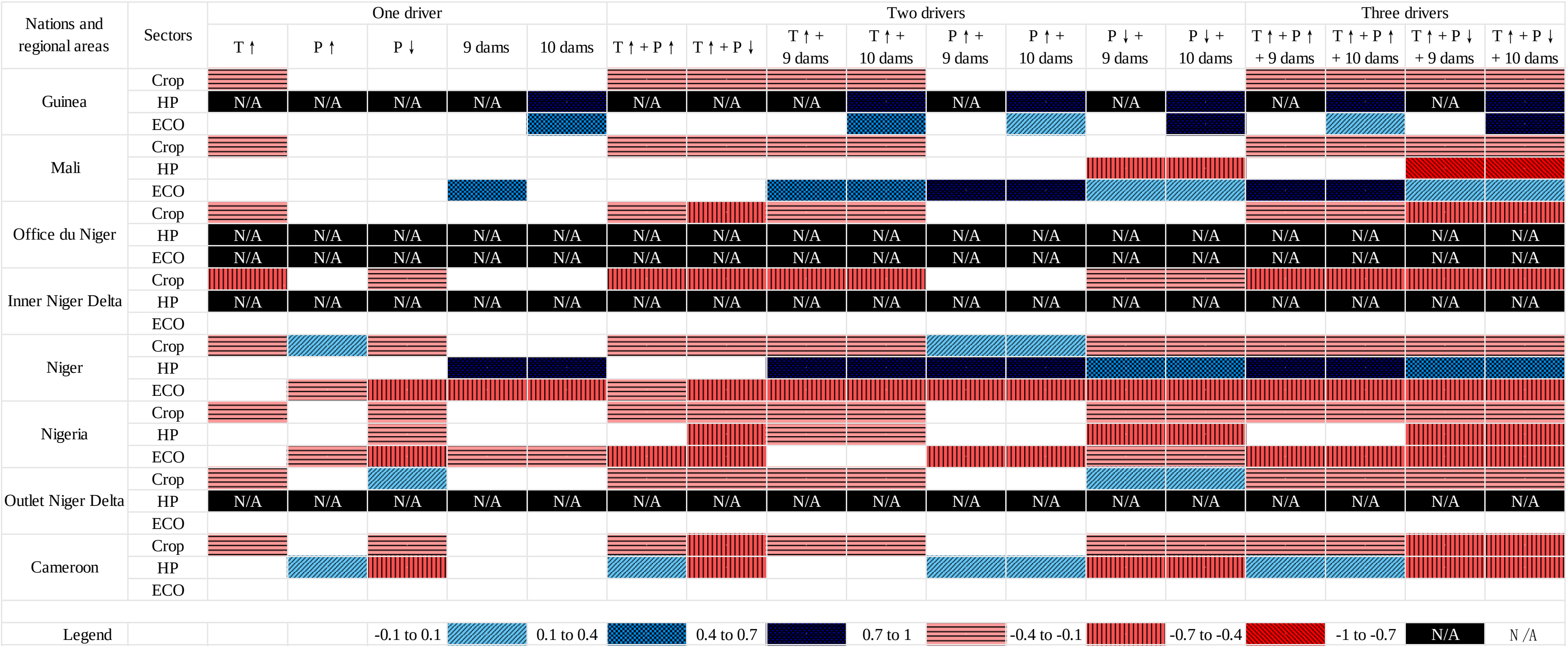

Figure S6. The joint effect of climate change and water infrastructure development on the maximum change of national and regional resilience (***SI₂***) of crop production, hydropower generation, and ecosystem health compared to the baseline scenario. Maximum index value changes between +0.1 and −0.1 are defined as "no change" (colored in white). Index value changes from 0.1 to 0.4, from 0.4 to 0.7, and above 0.7 are defined as "minor," "moderate," and "major" changes, respectively. Blue colors (from light to dark) are used to represent positive changes and red colors (from light to dark) are used to represent negative changes. Abbreviations: T, temperature; P, precipitation; crop, crop production; HP, hydropower generation; ECO, ecosystem health
