# Supplementary material for: Quantifying the Sustainability of Water Availability for the Water‐Food‐Energy‐Ecosystem Nexus in the Niger River Basin
Source: Earths Future. 2018 Sep 22;6(9):1292–310. doi: 10.1029/2018EF000923 (PMC6473704; doi:10.1029/2018EF000923)
Supplement: Supplementary file 2 — Table S1 [file EFT2-6-1292-s002.pdf]

AGU PUBLICATIONS

Earth's Future

Supporting Information for

Quantifying the Sustainability of Water Availability for the Water-Food-Energy-Ecosystem Nexus in the Niger River Basin

Jie Yang[1,2], Y. C. Ethan Yang[2*], Hassaan F. Khan[3], Hua Xie[4], Claudia Ringler[4], Andrew Ogilvie[5], Ousmane Seidou[6,7], Abdouramane Gado Djibo[8], Frank van Weert[9], and Rebecca Tharme[10]

[1.] State Key Laboratory of Eco-hydraulics in Northwest Arid Region of China (Xi'an University of Technology), Xi'an, 710048
[2.] Department of Civil and Environmental Engineering, Lehigh University, Bethlehem, PA 18015, USA
[3.] Department of Civil and Environmental Engineering, University of Massachusetts, Amherst, MA 01003, USA
[4.] International Food Policy Research Institute, Washington, DC, 20006, USA
[5.] IRD UMR G-EAU, BP 5095, 34196 Montpellier CEDEX 5, France
[6.] Department of Civil Engineering, University of Ottawa, Ottawa, ON, Canada
[7.] United Nations University, Institute for Water, Environment and Health, Hamilton, ON, Canada
[8.] Wetlands International, Bamako, Mali
[9.] Wetlands International, 6700 AL Wageningen, The Netherlands
[10.] Riverfutures, Cressbrook, Derbyshire, SK17 8SX, UK
[*] Corresponding author, +1-610-758-5685; yey217@lehigh.edu

Contents of this file

Table S1 to S3

Additional Supporting Information (Files uploaded separately)

None

Introduction

The supplement provide additional tables for the manuscript.

Table S1 - List of Regional Climate Models and General Circulation Models used in the analysis

**Regional climate model**	**CANRCM4**	**CCLM-4-8-17**	**HIRHAM5-v2**	**RACMO22T**	**RACMO22T**	**RCA4-v1**	**WRF331-v1**
**Institution (RCM)**	Canadian Centre for Climate Modelling and Analysis	Climate Limited-area Modelling Community	Danish Meteorological Institute	Royal Netherlands Meteorological Institute	Royal Netherlands Meteorological Institute	Swedish Meteorological and Hydrological Institute, Rossby Centre	Uni Research and the Bjerknes Centre for Climate Research
**Driving GCM**	**CanESM2**	**ICHEC-EC-EARTH**	**ICHEC-EC-EARTH**	**MOHC-HadGEM2-ES**	**ICHEC-EC-EARTH**	**CCCma-CanESM2**	**NCC-NorESM1-M**
**Institution (GCM)**	Canadian Centre for Climate Modelling and Analysis	Swedish Meteorological and Hydrological Institute	Swedish Meteorological and Hydrological Institute	UK Meteorological office	Swedish Meteorological and Hydrological Institute	Canadian Centre for Climate Modelling and Analysis	Norwegian Climate Center

Table S2 - Annual average irrigated crop production under different scenarios

Climate change		# of dams	Annual average irrigated crop production (ton/yr)								
Temp.	Precip.		Guinea	Mail	Office du Niger	Inner Niger Delta	Niger	Nigeria	Outlet Niger Delta	Cameroon	Whole river basin
+0 ℃	+0%	6	1224421	4064218	598734	1260597	2448950	26413510	2569285	1077815	35228914
+0 ℃	+0%	9	1224421	4126168	598734	1285921	2450495	26416426	2570996	1077815	35295324
+0 ℃	+0%	10	1224415	4105249	599252	1263499	2450495	26416348	2571000	1077815	35274322
+0 ℃	+15%	6	1190912	4090554	600582	1285174	2593764	26497216	2522200	1090731	35463176
+0 ℃	+15%	9	1190912	4112341	600582	1280627	2594769	26496864	2521852	1090731	35485617
+0 ℃	+15%	10	1190912	4113648	600922	1281606	2594769	26496864	2521852	1090731	35486925
+0 ℃	+30%	6	1196519	4085140	607064	1292520	2737325	26535058	2479662	1097636	35651677
+0 ℃	+30%	9	1196519	4099817	607064	1291618	2737878	26535058	2479662	1097636	35666908
+0 ℃	+30%	10	1196519	4100311	607078	1291824	2737878	26535058	2479662	1097636	35667401
+0 ℃	-15%	6	1255398	4084216	651939	1304528	2308292	26074198	2625890	1054336	34776438
+0 ℃	-15%	9	1255398	4110508	651939	1296020	2309858	26076142	2626356	1054336	34806240
+0 ℃	-15%	10	1255543	4115649	653504	1297385	2309865	26076297	2626361	1054336	34811689
+0 ℃	-30%	6	1350518	3871836	712077	1172851	2131262	25402859	2685465	1017437	33773912
+0 ℃	-30%	9	1350518	3879724	712077	1170053	2132137	25403294	2685465	1017437	33783110

+0 ℃	-30%	10	1350742	3876207	713437	1170834	2132127	25403294	2685465	1017437	33779808
+1.5 ℃	+0%	6	1290705	3791926	642595	1096066	2344013	25649543	2466849	1066661	34142849
+1.5 ℃	+0%	9	1290705	3806436	642595	1088612	2345427	25651882	2467813	1066661	34161112
+1.5 ℃	+0%	10	1290772	3806841	642661	1089272	2345429	25651787	2467813	1066661	34161489
+1.5 ℃	+15%	6	1284803	3836317	625476	1164381	2499095	25724799	2416413	1079210	34424225
+1.5 ℃	+15%	9	1284803	3845376	625476	1157782	2499920	25724241	2415825	1079210	34433550
+1.5 ℃	+15%	10	1284803	3846358	625746	1158317	2499920	25724241	2415825	1079210	34434532
+1.5 ℃	+30%	6	1281251	3815254	632455	1160353	2658215	25721939	2370243	1088592	34565251
+1.5 ℃	+30%	9	1281251	3824105	632455	1158469	2658738	25721939	2370243	1088592	34574624
+1.5 ℃	+30%	10	1281251	3825061	632605	1159018	2658738	25721939	2370243	1088592	34575581
+1.5 ℃	-15%	6	1354383	3707598	653813	1059125	2182217	25305079	2529782	1038640	33587916
+1.5 ℃	-15%	9	1354383	3717022	653813	1054334	2183259	25306875	2529835	1038640	33600179
+1.5 ℃	-15%	10	1354585	3719051	654779	1054174	2183249	25306877	2529782	1038640	33602402
+1.5 ℃	-30%	6	1445915	3470244	630237	965310	1995865	24576597	2591234	995707	32484328
+1.5 ℃	-30%	9	1445915	3473329	630237	963402	1996416	24576873	2591234	995707	32488239
+1.5 ℃	-30%	10	1446303	3472795	630750	963167	1996413	24576864	2591234	995707	32488083

+3 ℃	+0%	6	1339674	3361861	571316	866299	2199966	24832752	2364462	1052442	32786694
+3 ℃	+0%	9	1339674	3376966	571316	870735	2201046	24836066	2365712	1052442	32806193
+3 ℃	+0%	10	1339836	3377966	572016	870947	2201046	24835982	2365765	1052442	32807273
+3 ℃	+15%	6	1288360	3408552	579524	906620	2363898	24900786	2316682	1065295	33026892
+3 ℃	+15%	9	1288360	3422235	579524	912169	2364579	24899898	2315833	1065295	33040368
+3 ℃	+15%	10	1288360	3423187	579992	912543	2364579	24899898	2315833	1065295	33041320
+3 ℃	+30%	6	1296134	3410311	562508	952591	2537251	24910946	2266696	1074897	33229539
+3 ℃	+30%	9	1296134	3415257	562508	951194	2537679	24910937	2266687	1074897	33234904
+3 ℃	+30%	10	1296134	3416001	562590	951849	2537679	24910937	2266687	1074897	33235648
+3 ℃	-15%	6	1358034	3281583	581242	823314	2061344	24486129	2439302	1023442	32210533
+3 ℃	-15%	9	1358034	3285616	581242	821549	2062049	24486565	2438651	1023442	32215706
+3 ℃	-15%	10	1358243	3286308	581433	821956	2061987	24487211	2439297	1023442	32217191
+3 ℃	-30%	6	1421895	3092834	584885	735596	1889140	23751032	2505655	969713	31124613
+3 ℃	-30%	9	1421895	3093976	584885	734629	1889569	23746947	2505652	969713	31122100
+3 ℃	-30%	10	1422568	3093637	585437	733719	1889568	23746039	2505652	969713	31121525
+4.5 ℃	+0%	6	1247051	2949622	474724	656317	2080366	23991211	2256078	1034796	31303046

+4.5 ℃	+0%	9	1247051	2954842	474724	654913	2081223	23993553	2256703	1034796	31311465
+4.5 ℃	+0%	10	1247199	2955809	474951	655280	2081223	23993631	2256752	1034796	31312658
+4.5 ℃	+15%	6	1238199	3001063	482990	706867	2235725	24071426	2208441	1050414	31596827
+4.5 ℃	+15%	9	1238199	3005392	482990	705610	2236305	24071602	2208587	1050414	31601912
+4.5 ℃	+15%	10	1238199	3006106	483198	705881	2236305	24071602	2208587	1050414	31602626
+4.5 ℃	+30%	6	1246786	3036755	488427	752003	2394637	24089280	2166682	1060248	31827705
+4.5 ℃	+30%	9	1246786	3040873	488427	751374	2395012	24088919	2166261	1060248	31831837
+4.5 ℃	+30%	10	1246786	3041087	488496	751524	2395012	24088919	2166261	1060248	31832051
+4.5 ℃	-15%	6	1253419	2838690	462762	598744	1951445	23677813	2339047	1001575	30722941
+4.5 ℃	-15%	9	1253419	2841932	462762	598279	1951874	23678346	2338585	1001575	30727145
+4.5 ℃	-15%	10	1253681	2842555	463012	598608	1951861	23678167	2338585	1001575	30727840
+4.5 ℃	-30%	6	1240992	2636743	445183	517041	1798789	22973624	2408915	939739	29589887
+4.5 ℃	-30%	9	1240992	2637780	445183	516759	1799153	22966129	2408915	939739	29583793
+4.5 ℃	-30%	10	1241394	2638282	445490	516814	1799127	22966129	2408915	939739	29584672

Table S3 - Annual average hydropower generation under different scenarios

Climate change		# of dams	Annual average hydropower generation (GWh/yr)								
Temp.	Precip.		Guinea	Mail	Office du Niger	Inner Niger Delta	Niger	Nigeria	Outlet Niger Delta	Cameroon	Whole river basin
+0 °C	+0%	6	N/A	426	N/A	N/A	N/A	112614	N/A	5430	118469
+0 °C	+0%	9	N/A	29440	N/A	N/A	13941	112456	N/A	5430	161267
+0 °C	+0%	10	53996	29443	N/A	N/A	13941	112577	N/A	5430	215386
+0 °C	+15%	6	N/A	437	N/A	N/A	N/A	137542	N/A	8819	146799
+0 °C	+15%	9	N/A	29614	N/A	N/A	14025	142036	N/A	8819	194495
+0 °C	+15%	10	53996	29618	N/A	N/A	14025	142036	N/A	8819	248494
+0 °C	+30%	6	N/A	447	N/A	N/A	N/A	145764	N/A	12038	158249
+0 °C	+30%	9	N/A	29668	N/A	N/A	14025	147031	N/A	12038	202762
+0 °C	+30%	10	53996	29673	N/A	N/A	14025	147031	N/A	12038	256763
+0 °C	-15%	6	N/A	414	N/A	N/A	N/A	98868	N/A	3174	102457
+0 °C	-15%	9	N/A	26677	N/A	N/A	10820	92028	N/A	3174	132699
+0 °C	-15%	10	53992	26718	N/A	N/A	10857	92442	N/A	3174	187184
+0 °C	-30%	6	N/A	402	N/A	N/A	N/A	76730	N/A	1828	78960
+0 °C	-30%	9	N/A	16564	N/A	N/A	4688	70278	N/A	1828	93358
+0 °C	-30%	10	28131	16864	N/A	N/A	4637	70412	N/A	1828	121872
+1.5 °C	+0%	6	N/A	423	N/A	N/A	N/A	110453	N/A	5127	116003
+1.5 °C	+0%	9	N/A	29274	N/A	N/A	13763	108866	N/A	5127	157031
+1.5 °C	+0%	10	53996	29278	N/A	N/A	13770	109114	N/A	5127	211285
+1.5 °C	+15%	6	N/A	435	N/A	N/A	N/A	136242	N/A	8457	145134
+1.5 °C	+15%	9	N/A	29599	N/A	N/A	14025	140444	N/A	8457	192525
+1.5 °C	+15%	10	53996	29602	N/A	N/A	14025	140444	N/A	8457	246523
+1.5 °C	+30%	6	N/A	445	N/A	N/A	N/A	143950	N/A	11633	156029
+1.5 °C	+30%	9	N/A	29662	N/A	N/A	14025	145484	N/A	11633	200804
+1.5 °C	+30%	10	53996	29666	N/A	N/A	14025	145484	N/A	11633	254804
+1.5 °C	-15%	6	N/A	413	N/A	N/A	N/A	96649	N/A	3059	100121
+1.5 °C	-15%	9	N/A	25887	N/A	N/A	10179	88570	N/A	3059	127695

+1.5 ℃	-15%	10	53972	25978	N/A	N/A	10207	88736	N/A	3059	181953
+1.5 ℃	-30%	6	N/A	401	N/A	N/A	N/A	74581	N/A	1779	76760
+1.5 ℃	-30%	9	N/A	15377	N/A	N/A	4254	68261	N/A	1779	89671
+1.5 ℃	-30%	10	24906	15576	N/A	N/A	4170	68317	N/A	1779	114749
+3 ℃	+0%	6	N/A	422	N/A	N/A	N/A	108387	N/A	4912	113721
+3 ℃	+0%	9	N/A	29137	N/A	N/A	13614	105531	N/A	4912	153194
+3 ℃	+0%	10	53996	29117	N/A	N/A	13604	105702	N/A	4912	207331
+3 ℃	+15%	6	N/A	433	N/A	N/A	N/A	134837	N/A	8172	143442
+3 ℃	+15%	9	N/A	29588	N/A	N/A	14025	138530	N/A	8172	190315
+3 ℃	+15%	10	53996	29590	N/A	N/A	14025	138530	N/A	8172	244313
+3 ℃	+30%	6	N/A	443	N/A	N/A	N/A	142743	N/A	11304	154490
+3 ℃	+30%	9	N/A	29653	N/A	N/A	14025	143878	N/A	11304	198860
+3 ℃	+30%	10	53996	29657	N/A	N/A	14025	143878	N/A	11304	252859
+3 ℃	-15%	6	N/A	411	N/A	N/A	N/A	94524	N/A	2962	97897
+3 ℃	-15%	9	N/A	25306	N/A	N/A	9373	85556	N/A	2962	123197
+3 ℃	-15%	10	53482	25298	N/A	N/A	9394	85813	N/A	2962	176949
+3 ℃	-30%	6	N/A	400	N/A	N/A	N/A	72335	N/A	1739	74473
+3 ℃	-30%	9	N/A	14049	N/A	N/A	3899	64226	N/A	1739	83913
+3 ℃	-30%	10	23917	14131	N/A	N/A	3813	64229	N/A	1739	107829
+4.5 ℃	+0%	6	N/A	420	N/A	N/A	N/A	106482	N/A	4707	111609
+4.5 ℃	+0%	9	N/A	28961	N/A	N/A	13239	102059	N/A	4707	148967
+4.5 ℃	+0%	10	53996	28937	N/A	N/A	13301	102324	N/A	4707	203266
+4.5 ℃	+15%	6	N/A	431	N/A	N/A	N/A	133086	N/A	7874	141392
+4.5 ℃	+15%	9	N/A	29576	N/A	N/A	14025	136719	N/A	7874	188195
+4.5 ℃	+15%	10	53996	29578	N/A	N/A	14025	136749	N/A	7874	242223
+4.5 ℃	+30%	6	N/A	441	N/A	N/A	N/A	141471	N/A	10971	152884
+4.5 ℃	+30%	9	N/A	29645	N/A	N/A	14025	142268	N/A	10971	196910
+4.5 ℃	+30%	10	53996	29649	N/A	N/A	14025	142268	N/A	10971	250909
+4.5 ℃	-15%	6	N/A	410	N/A	N/A	N/A	92378	N/A	2873	95660
+4.5 ℃	-15%	9	N/A	24787	N/A	N/A	8651	82984	N/A	2873	119294
+4.5 ℃	-15%	10	53128	24816	N/A	N/A	8621	83087	N/A	2873	172525
+4.5 ℃	-30%	6	N/A	399	N/A	N/A	N/A	69570	N/A	1709	71677

+4.5 ℃	-30%	9	N/A	13111	N/A	N/A	3627	60125	N/A	1709	78572
+4.5 ℃	-30%	10	23173	13062	N/A	N/A	3546	60170	N/A	1709	101659
